# Exploring the Isoreticular Continuum between Phosphonate- and Phosphinate-Based Metal–Organic Frameworks

**DOI:** 10.1021/acs.inorgchem.2c03271

**Published:** 2022-11-11

**Authors:** Soňa Ondrušová, Matouš Kloda, Jan Rohlíček, Marco Taddei, Jan K. Zaręba, Jan Demel

**Affiliations:** † Institute of Inorganic Chemistry of the Czech Academy of Sciences, 250 68 Řež, Czech Republic; ‡ Faculty of Science Charles University, 128 00 Praha 2, Czech Republic; § Department of Structure Analysis, Institute of Physics, Czech Academy of Sciences, Prague 18221, Czech Republic; ∥ Department of Chemistry and Industrial Chemistry, University of Pisa, Via Giuseppe Moruzzi, 13, Pisa 56124, Italy; ⊥ Institute of Advanced Materials, Wrocław University of Science and Technology, Wybrzeże, Wyspiańskiego 27, Wrocław 50-370, Poland

## Abstract

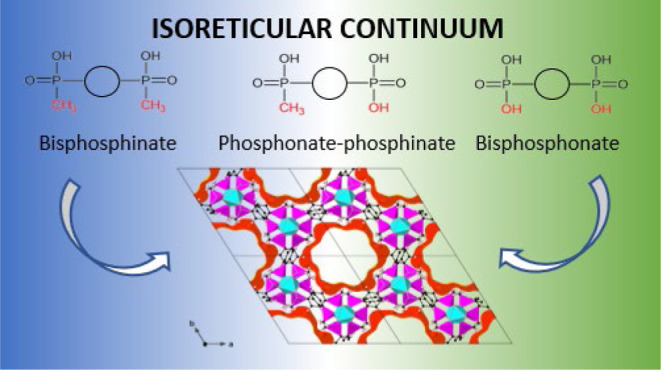

The rational design of metal–organic frameworks (MOFs) is one of the driving forces behind the great success that this class of materials is experiencing. The so-called isoreticular approach is a key design tool, very often used to tune the size, steric properties, and additional functional groups of the linker used. In this work, we go one step further and show that even linkers with two different coordinating groups, namely, phosphonate and phosphinate, can form isoreticular MOFs. This effectively bridges the gap between MOFs utilizing phosphinate and phosphonate coordinating groups. Using a novel bifunctional ligand, 4-[hydroxy(methyl)phosphoryl]phenylphosphonic acid [H_3_PPP(Me)], we were able to prepare ICR-12, a MOF isoreticular to already published MOFs containing bisphosphinate linkers (e.g., ICR-4). An isostructural MOF ICR-13 was also successfully prepared using 1,4-benzenediphosphonic acid. We envisage that this strategy can be used to further enlarge the pool of MOFs.

## Abstract 2

We describe the isoreticular continuum across MOF classes with different functional groups: purely phosphonate, purely phosphinate, and mixed phosphonate−phosphinate. Structural and physicochemical studies of phosphonato-phosphinate ICR-12 show that this compound is truly the middle ground between bisphosphinate-based ICR-2 and bisphosphonate-based ICR-13 “boundary” structures.

## Introduction

Metal–organic frameworks (MOFs) are porous coordination polymers consisting of inorganic nodes connected by organic linkers. These materials show chemical variability and topological diversity which predestines them to diverse applications, for example, gas storage and separation,^1^ catalysis,^2,3^ sensing,^4^ and medicinal applications.^5^ However, even after more than 2 decades of research, MOF stability represents a challenge, especially when it comes to applications in an aqueous environment.^6^ Stability of MOFs is, in a large part, determined by the strength of the coordination bond between the linkers and metal centers. Ligands that are considered soft according to Pearson’s theory of hard and soft acids and bases (HSAB),^7^ such as linkers based on imidazole or bipyridine, are often used in combination with divalent metals such as Zn^2+^, Cu^2+^, or Co^2+^. This can lead to MOFs stable in alkaline conditions.^8^ This class of materials, however, makes up only a relatively small part of all known MOF structures. The majority of MOFs are prepared using carboxylate linkers. These are readily accessible by established synthetic routes, bind to metal ions of all valences, and produce predictable MOF structures which are often highly porous.^9^ Unfortunately, carboxylate MOFs usually do not show high stability, especially toward water.^10,11^ One of the strategies to increase the MOF stability is to use linkers that form stronger bonds to the metal centers. According to the HSAB theory, the phosphonate group has greater affinity to hard metal ions such as Zr^4+^, Al^3+^, and so forth which makes the resulting structures more chemically and thermally robust.^12^

Metal phosphonates have not gained as much publicity as carboxylates so far, nonetheless in recent years, growing number of porous structures utilizing phosphonate linkers is being reported. Examples include zeolite-like frameworks using alkylphosphonates,^13,14^ MOFs using alkyl- and aryl-bisphosphonates with varying spacer length,^15−17^ or nonlinear linkers such as tri- and tetra-topic arylphosphonates.^18^ The use of a phosphonate group for construction of MOFs remains, however, a double-edged sword—on the one hand, there is much desired merit of stability of metal phosphonate bonding, but on the other hand, the coordination chemistry is complex because the phosphonate group strongly binds with great variability through all three accessible oxygen atoms.^19,20^ Indeed, fully deprotonated phosphonate can theoretically participate in as much as 16 distinct coordination modes,^21^ which translates to the poor predictability of phosphonate MOF structures; extensive metal–oxygen binding is also responsible for preferred formation of nonporous, layered structures.^19^

**Figure 1:**
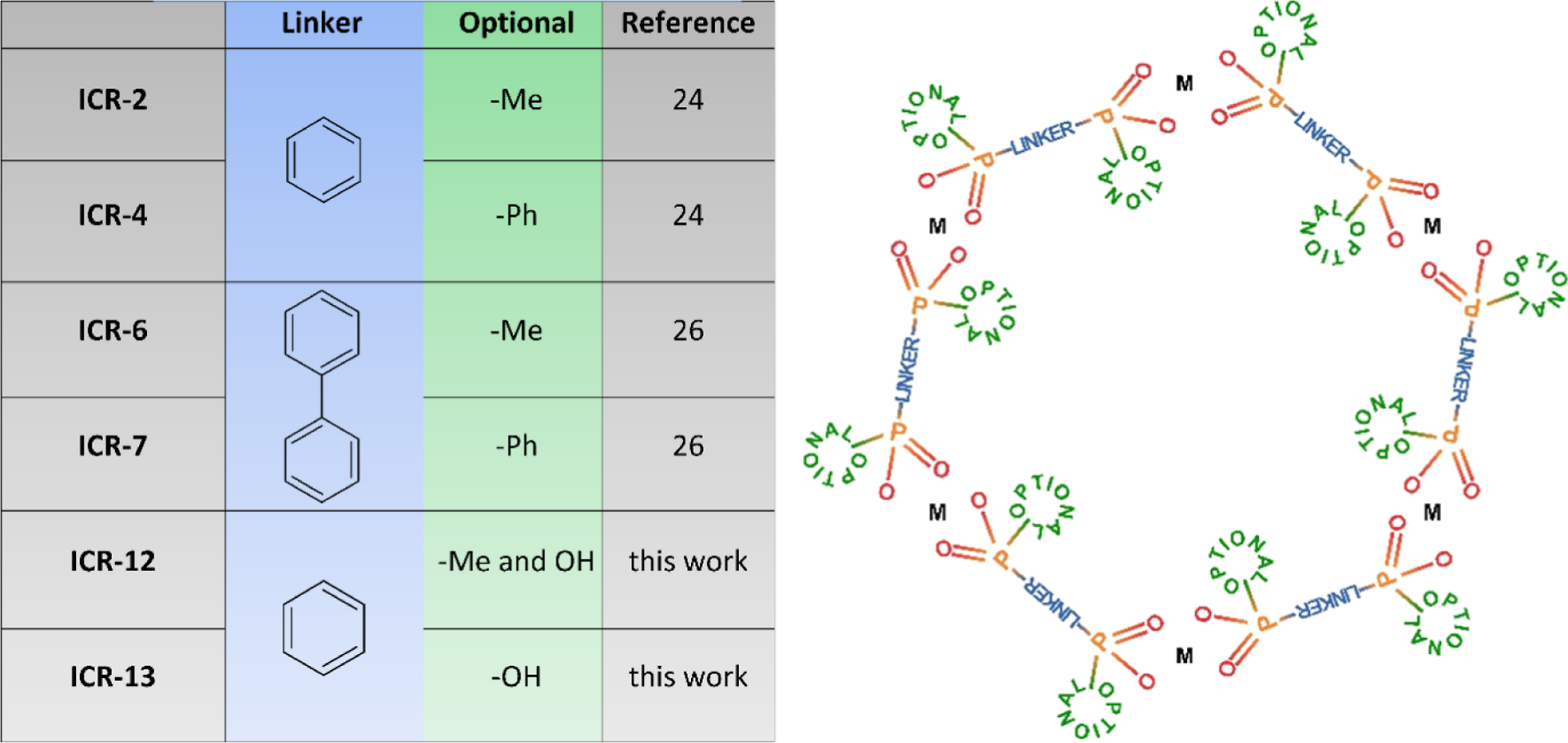
Versatility of phosphinate linkers in the isoreticular series of MOFs.

One way to circumvent the limitations of the phosphonate group is the use of phosphonate monoesters.^22^ By substituting one of the oxygen atoms in the phosphonate group with an alkoxy group, coordination modes closer to those of carboxylates are achieved. Furthermore, the alkoxy group provides the ligands with some degree of structural tunability. However, the ester bond is prone to hydrolysis, which limits the options for synthesis conditions. Another way to restrict the coordination modes of the phosphonate group is synthesis under controlled pH, allowing only single deprotonation of the phosphonate group.^23^ The same coordination modes, but without the danger of hydrolysis or need for restricted reaction conditions, can be achieved by alkyl- or aryl-phosphinate binding groups. The most common coordination motif in phosphinate coordination polymers, eight-membered M-O-P-O-M-O-P-O rings,^24^ was indeed identified as a prevalent motif in phosphonate MOFs as well.^18^ In terms of acidity and HSAB theory, phosphinic acids fall between carboxylic and phosphonic acids. Recently, we have shown that linkers based on phosphinic acids can serve as an alternative to carboxylate and phosphonate linkers for the synthesis of MOFs.^25^ The main advantage of phosphinate linkers over carboxylates is a stronger bond with trivalent metal centers, which results in increased MOF stability.^26^ Importantly, the isoreticular design, often utilized in carboxylate MOF chemistry, is applicable to metal phosphinates as well.^27^ This enables the targeted design of phosphinate-based MOFs by the selection or modification of the starting ligands. In particular, the “P-optional” group (Figure 1) in metal phosphinates provides a more versatile tool for tuning the properties of the ligands compared to the alkoxy group in phosphonate monoesters. Based on the above, isoreticular design could be applied not only between phosphinates with different substituents but also between phosphinates and phosphonate monoesters or singly deprotonated phosphonates.

The combination of two or more types of binding groups in one linker is an established strategy in MOF synthesis.^28^ This crystal engineering approach is promising because the introduction of an additional functional group can lead to extended functionalities, novel topologies, and enhanced chemical properties. In the case of phosphonate MOFs, the most commonly used additional coordination group is the carboxylate group.^29−31^ Alternatively, the coordination of an additional sulfonate group can lead to the formation of 3D coordination polymers with layered structures.^32^ High stability of phosphonate MOFs can also be combined with the tunability of bridging pyridyl groups.^33^ In the field of metal phosphinates, the phosphinate ligands bearing another coordinating group yielded almost exclusively non-porous coordination polymers. In these materials, the phosphinic group was primarily combined with the carboxylic group,^34,35^ nevertheless, auxiliary N-donating groups were also reported.^36,37^

It should be noted, however, that exchanging one or more of the binding groups on the ligand generally leads to different topologies of the resulting structure. A notable exception is the combination of carboxylate and tetrazole binding groups in rare earth metal MOFs.^38^

In recent years, it was demonstrated that the isoreticular chemistry concept is not limited merely to carboxylate MOFs, but can be also extended to other non-carboxylate classes, as demonstrated for metal phosphonates^18,39,40^ and phosphinates.^25^ If each of those material classes is capable of isoreticular extension/modification of their structures, it raises a question whether molecular building blocks comprising distinct phosphonate and phosphinate functional groups are likely to feature the same self-assembly property. Accordingly, in this contribution we target a fundamental understanding of the properties and provide a proof of concept of isoreticular design extending between phosphonates and phosphinates. To this end, we introduce, for the first-time, an organic ligand combining both these binding groups, that is, 4-[hydroxy(methyl)phosphoryl]phenylphosphonic acid [H_3_PPP(Me)], which is a logical middle ground between archetypal bisphosphinate and bisphosphonate linkers. By reaction with Fe^3+^ salt we have obtained a new MOF named ICR-12, isoreticular to all-phosphinate MOF ICR-2,^25^ which is notably a first example of metal phosphonato-phosphinates. It turns out that by employing similar synthetic conditions, 1,4-benzenediphosphonic acid also forms a new phosphonate Fe^3+^ MOF (ICR-13), again showing isoreticularity to ICR-2 and ICR-12 MOFs. Thus, this work demonstrates the isoreticular continuum overarching not only conventional metal phosphonates and phosphinates but also unprecedented class of metal phosphonato-phosphinates.

## Results and Discussion

### Synthesis

**Figure 2:**
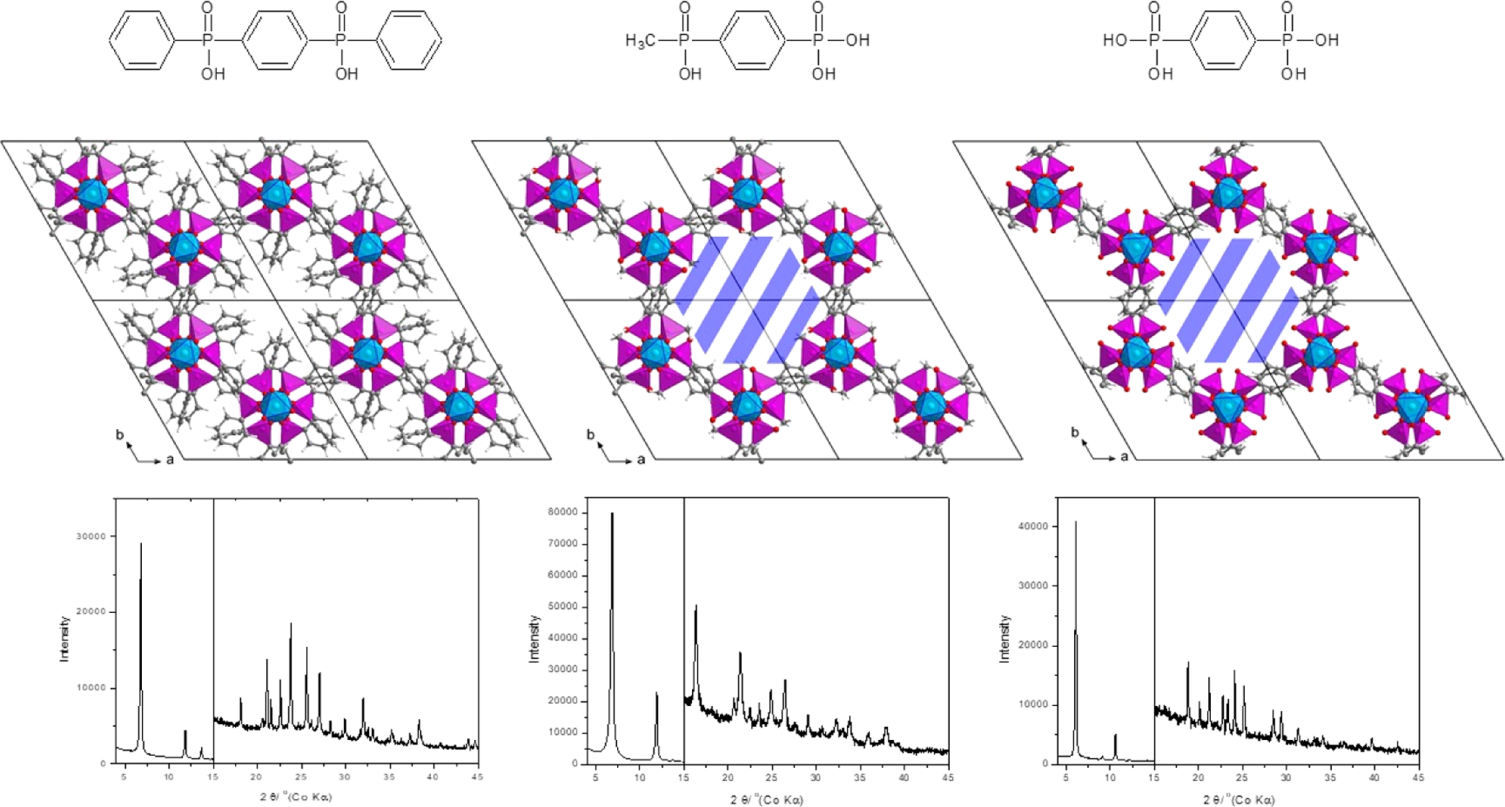
Comparison of ligands (top), view of the structure along the *c* axes (middle), and PXRD patterns of ICR-4 (left), ICR-12 (middle), and ICR-13 (right).

In order to confirm the isoreticular continuum between metal phosphinates and phosphonates we have synthesized 4-[hydroxy(methyl)phosphoryl]phenylphosphonic acid [H_3_PPP(Me), see Figure 2 middle top]. We employed an analogous procedure as was used for the preparation of phenylene-1,4-bis-methylphosphinic acid [H_2_PBP(Me)],^25^ that is, coupling reaction of diethyl (4-bromophenyl)phosphonate with methyl methylphosphinate and subsequent ester deprotection using Me_3_SiBr.

The reaction of H_3_PPP(Me) and 1,4-benzenediphosphonic acid with FeCl_3_ at high temperatures gave microcrystalline powders with very similar powder X-ray diffraction (PXRD) patterns, see Figure 2. The phases were denoted ICR-12 [H_3_PPP(Me) ligand] and ICR-13 (1,4-benzenediphosphonic ligand). The reaction between H_3_PPP(Me) and FeCl_3_·6H_2_O occurs similarly to the synthesis of ICR-2 from H_2_PBP(Me) using ratio 2:1 in anhydrous ethanol. The reaction was carried out in a PTFE-lined stainless-steel autoclave at 120 °C giving acicular crystals. In the PXRD pattern, the low angle reflections of ICR-12 are very close to those of ICR-2 (Figure S16). However, unlike most of the MOFs of ICR series, which crystallize in the trigonal system, ICR-2 crystallizes in the monoclinic system. For this reason, the structure of ICR-12 was determined based on the ICR-4 which uses H_2_PBP(Ph) as the linker (Figure 2 left). Raising the temperature to 250 °C or changing solvent to DMF leads to an identical phase with lower crystallinity. Using AlCl_3_ instead of FeCl_3_ also leads to an identical phase with a small amount of unknown impurity (Figure S17).

In the case of 1,4-benzenediphosphonic acid, a variation of the synthetic route was required. Dry mixture of the reactants was first treated in a ball mill for 30 min, followed by solvothermal crystallization in anhydrous ethanol at 250 °C. The resulting phase was named ICR-13, and the PXRD pattern of this phase is again similar to ICR-2 and ICR-12. With milling omitted, a poorly reproducible mixture of non-porous phases was produced instead for both Fe^3+^ and Al^3+^. Despite multiple Al benzenediphosphonates being reported in the literature,^41,42^ no conclusive match to a known phase was found. Repeating the synthetic procedure with milling included using AlCl_3_ instead of FeCl_3_ resulted in a mixture of ICR-13 with another unknown phase (Figure S18).

### Structure Determination

**Figure 3:**
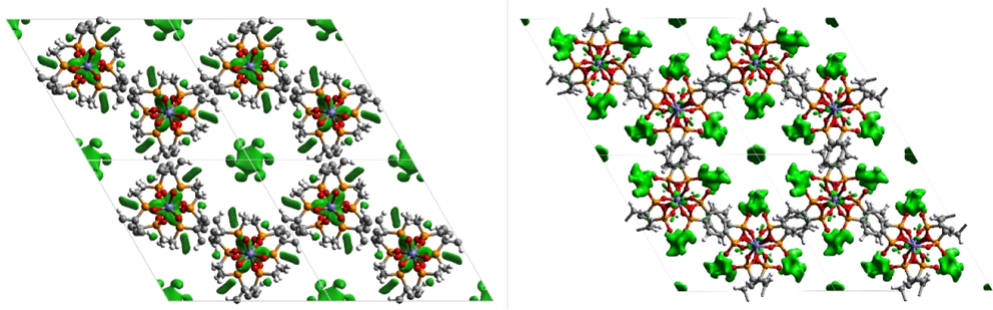
Partially solved crystal structures of ICR-12 (left) and ICR-13 (right) showing the honeycomb structure and relatively large pores. The green objects inside the pores represent the difference Fourier with the positive value at the 2σ level. This indicates the presence of additional atomic or molecular structures in the pores.

Both ICR-12 and ICR-13 form isoreticular scaffold as ICR-4; however, there is an indication that significant electron densities are present in the middle of the pores in both cases. The Rietveld refinement revealed a residual electron density of an unidentified shape facing or occupying the center of the pore, see Figure 3. Unfortunately, the quality of the PXRD data did not allow the description of the observed residual electron density and this part of both crystal structures remains unclear. Despite this fact, the PXRD data allowed us to confirm the main structural motif of both MOFs creating the honeycomb arrangement of linkers coordinating the Fe atoms, which is similar to ICR-4. The asymmetric unit of both structures consists of two symmetrically independent halves of the ligand and two Fe^3+^ cations located on the threefold axis (Figures S10 and S11, Tables S2 and S3), which corresponds to the structure of ICR-4. The attached XYZ files represent the atomic models of compounds ICR-12 and ICR-13, which were used to simulate PXRD patterns during Rietveld refinement. The quality of the measured PXRD data did not allow a complete description of their structures and some parts of their crystal structures remain unclear. In particular, parts of the crystal structures facing the pores were not fully described or not described at all. These models were also used to calculate difference Fourier maps, the results of which are discussed in detail in this paper. For a detailed discussion of the crystal structure determination, see the Supporting Information.

Both newly prepared MOFs are isostructural and contain the same structural motifs seen not only in ICR-4, but in all of the Fe^3+^ based MOFs of the ICR family. This confirms the expected link between phosphonate and phosphinate MOF chemistry—the protonated OH group in phosphonates indeed allows isoreticular synthesis in the same way as the P-optional group in phosphinates. Thus, phosphinic acid linkers can be expected to introduce specific functional groups and to fine-tune the environment on phosphorus atoms in known phosphonate MOF structures. Using the opposite approach, phosphonate linkers can introduce free OH groups into known phosphinate structures, for example, to introduce ion exchange properties or enhance proton conductivity.

### Characterization

**Figure 4:**
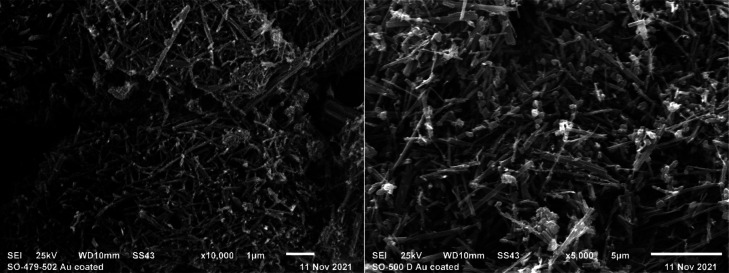
SEM images of ICR-12 crystals (left) and ICR 13 crystals (right).

The morphology of both MOFs was monitored by scanning electron microscopy (SEM). Similarly to ICR-2, both novel MOFs form rod-like crystals of inhomogeneous size with the crystals of ICR-13 being significantly bigger than ICR-12 (Figure 4).

**Figure 5:**
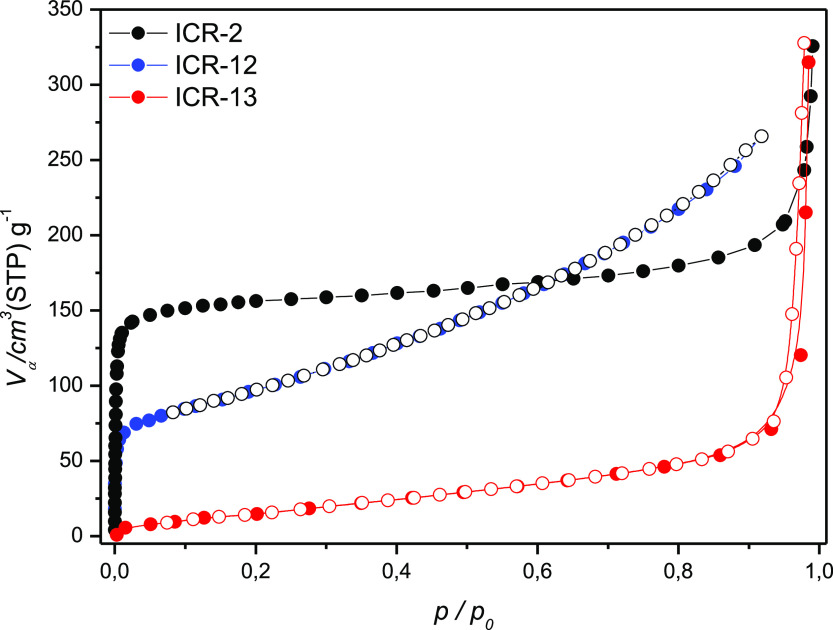
Nitrogen adsorption isotherms of as-synthesized ICR-2, ICR-12, and ICR-13.

The porosity of ICR-12 and ICR-13 was probed by measuring the N_2_ adsorption isotherms at 77 K (Figure 5, tabular data in Tables S4 and S5). In the case of ICR-12, measured isotherm corresponds to a microporous material. Calculation from PoreBlazer^43^ (assuming empty channels) suggested a specific surface area 819 m^2^ g^–1^, close to the value observed for ICR-2, see Table 1. The measured specific surface area of 396 m^2^ g^–1^, however, is significantly lower than the theoretical value or corresponding ICR-2 (MOF based on a bisphosphinate linker). In the case of ICR-13, no porosity was observed. This supports the observations from structure determination that the introduction of phosphonic group with higher connectivity leads to filling of pores.

**Table 1:** Specific Surface Areas, Pore Diameters, Calculated Pore Limiting Diameters, and Accessible Surface Areas for ICR-12 and ICR-13 Compared to ICR-2

sample	linker	specific surface area (m^2^ g^–1^)	pore diameter (nm)^b^	pore volume (cm^3^ g^–1^)	accessible surface area (m^2^ g^–1^)^c^	pore limiting diameter (nm)^c^	pore accessible volume (cm^3^ g^–1^)^c^
ICR-2^a^	H_2_PBPA(Me)	906	0.71	0.39	850	0.90	0.48
ICR-12	H_3_PPPA(Me)	396	0.86	0.57	819	0.88	0.33
ICR-13	1-4-bisphenylphosphonic acid	42	n.a.	n.a.	853	0.94	0.34

a Values taken from ref (27).

b Median pore diameter, for pore size distribution see Figure S21.

c Calculated by PoreBlazer software assuming empty channels.

CHN elemental analysis of ICR-13 also shows higher than expected amount of C, which can be explained by molecules of ligand and remaining solvent held by hydrogen bonds inside the pores. In the case of ICR-12, the observed amount of C is close to the calculated one. The CHN analyses of both products are summed up in Table 2. The sorption experiments, however, indicate that the pores are effectively blocked from gas access. This can be explained by the presence of additional Fe^3+^ cations and linker anions in the same ratio as in the structure, corresponding to their respective charges. In an attempt to clear the blocked pores, ICR-12 and ICR-13 were treated with 3 M NaOH solution in EtOH at room temperature. Unfortunately, the treatment with base almost immediately leads to color change from white to rusty. In the case of ICR-12 the sample lost its crystallinity, see Figure S19, whereas in the case of ICR-13, the PXRD shows the formation of a new unknown phase, see Figure S20. Trying less harsh conditions (0.05 M KOH) for 48 h similarly to the procedure described by Vilela et al.^44^ led to the sample destruction.

**Table 2:** Measured and Calculated (in Brackets) Mass Representation of Elements in ICR-12 and ICR-13

sample	formula^a^	H^b^	C^b^	P^c^	Fe^c^
ICR-12	Fe_2_(C_7_H_8_O_5_P_2_)_3_	3.30(2.98)	31.85(30.99)	22.00(21.70)	13.72(13.35)
ICR-13	Fe_2_(C_6_H_6_O_6_P2)_3_	3.27(2.21)	32.43(26.37)	22.67(19.19)	13.62(12.43)

a The formulas are based on Fe_2_(linker)_3_ ratio.

b Data obtained by CHN elemental analysis.

c Data obtained by EDX.

**Figure 6:**
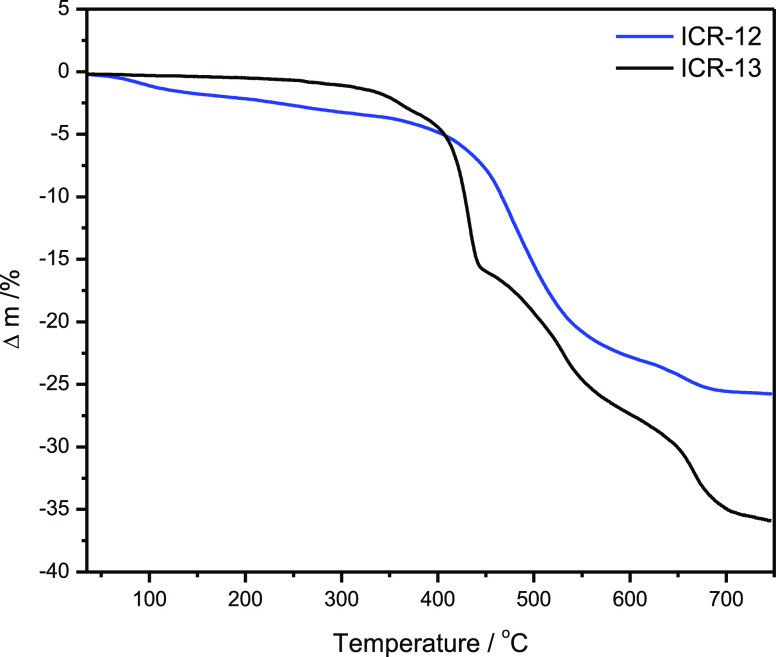
TG curves for ICR-12 and 13 in air.

Thermal stability in air of prepared MOFs was determined by the thermogravimetric analyses in conjunction with differential thermal analyses and mass spectroscopy (TGA/DTA/MS) (Figures 6, S14, and S15). Similarly, as other Fe^3+^ phosphinate-based MOFs, ICR-12 starts to decompose at 470 °C and ICR-13 at 435 °C (Figure 6). Only small amounts of solvents are desorbed prior to the framework decomposition (below 5% up to 300 °C).

## Conclusions

The principal objective of this work was to provide proof of concept for the isoreticular continuum across MOF classes with different functional groups: purely phosphonate, purely phosphinate, and mixed phosphonate–phosphinate. For this purpose, we synthesized a novel ligand, 4-[hydroxy(methyl)phosphoryl]phenylphosphonic acid. To the best of our knowledge, it is the first ligand combining phosphinate and phosphonate coordinating groups used for the construction of a MOF structure. The set of linkers was used for the preparation of two new Fe^3+^ MOFs, denoted ICR-12 and ICR-13, based on phenylene-1-phosphonic-4-methyl-phosphinic acid and 1,4-phenylene(bisphosphonic acid), respectively. Both MOFs are isostructural and crystallize in a trigonal space group with a honeycomb array of parallel channels, similarly to previously described phosphinate MOF ICR-4. Structural and physicochemical studies of phosphonato-phosphinate ICR-12 show that this compound is truly the middle ground between bisphosphinate-based ICR-2 and bisphosphonate-based ICR-13 “boundary” structures. Indeed, the specific surface area for ICR-12 (396 m^2^ g^–1^) is about half of that for ICR-2 phosphinate (906 m^2^ g^–1^) and no porosity was observed for ICR-13; the crystallographic results also show the presence of electron densities, which correspond to molecular species completely (ICR-13) and partially (ICR-12) blocking the pores. Increasing blockage inside the pores from ICR-12 to ICR-13, likely caused by the abundance of coordinating groups on the pore walls, is therefore the reason limiting the gas sorption property. In essence, the presented set of MOFs demonstrates the isoreticular continuum of physicochemical properties, which can be obtained for ligands with similar yet distinct functional groups.

Ultimately, the newly prepared phosphonate and phosphonate–phosphinate MOFs bear structural similarities to the entire family of Fe^3+^-based ICR MOFs. Thus, we provide the first proof of isoreticular synthesis extending from phosphinate to phosphonate linkers, which opens the door to the utilization of unique properties of phosphinates in known phosphonate MOF structures and vice versa.

## Experimental Procedures

### Materials

MePCl_2_, trimethylsilyl bromide (both Acros Organics), benzene (anhydrous), Pd(PPh_3_)_4_, FeCl_3_·6H_2_O (all Sigma-Aldrich), ethanol anhydrous (Fischer Chemical), 1,4-benzenediphosphonic acid (Epsilon Chimie), and Merrifield resin HL (Merck) were used as purchased. Acetonitrile and 1,4-dioxane (water-free, VWR Chemicals) and dichloromethane (HPLC grade, Fisher Scientific) were dried using solvent purification system SP-1 (LC technology). Triethylamine (Sigma-Aldrich) was freshly distilled from Na under Ar. The synthesis of linkers was performed under Ar using standard Schlenk techniques. Column chromatography was performed using Sigma-Aldrich 60 (70–230 mesh, 60 Å) silica gel. Preparation of diethyl (4-bromophenyl)phosphonate was performed as per literature protocol.^45^

#### Preparation of Methyl Methylphosphinate

A Schlenk tube was charged with 10 g (85 mmol) of MePCl_2_, three times evacuated and flushed with Ar, and diluted with 80 mL of dry benzene. In a second Schlenk tube, 8.4 mL (208 mmol) of dry MeOH were mixed with 11.9 mL (85 mmol) of dry triethylamine under an Ar atmosphere. The mixture was cooled by an ice bath and the solution of MePCl_2_ was slowly added. The formed precipitate was filtered off and the filtrate was evaporated, yielding an oily product which was directly used for further synthesis. For NMR spectra of the product, see the Supporting Information.

Yield: 10.7 g (80%).

#### Preparation of Diethyl 4-[Methoxy(methyl)phosphoryl]phenylphosphonate

A flask was charged with 5.1 g of Pd(PPh_3_)_4_ (4.4 mmol) and 12.8 g of diethyl (4-bromophenyl)phosphonate (43.7 mmol), three times evacuated and flushed with Ar, and diluted with 250 mL of dry dioxane. 6.2 g of methyl methylphosphinate (56 mmol) and dry triethylamine (7.1 mL) were added. The resulting mixture was stirred at 60 °C for 96 h. After cooling down to room temperature, the formed precipitate was filtered off and the filtrate was evaporated to dryness. The solid residue was purified by chromatography in dichloromethane. The crude product was then dissolved in 30 mL of acetone and 2.5 g of Merifield resin and 1.5 g of NaI were added. The mixture was stirred overnight, filtered, and washed with THF, water, and acetone. The resulting orange oil was characterized by NMR (see the Supporting Information) and directly used for the next step.

#### Preparation of 4-[Hydroxy(methyl)phosphoryl]phenylphosphonic Acid

A Schlenk tube was charged with 15.3 g of diethyl 4-[methoxy(methyl)phosphoryl]phenylphosphonate (56 mmol), evacuated, and flushed with argon three times, and then, 250 mL of dry acetonitrile was added followed by a dropwise addition of 29 mL of trimethylsilyl bromide (212 mmol). The resulting mixture was stirred at 40 °C overnight. After cooling down, the solution was evaporated to dryness. The solid residue was dissolved in water and the water solution was washed with diethyl ether three times. The water fraction was evaporated and the crude product was purified by trituration with acetone. The resulting white powder was characterized by NMR (see the Supporting Information).

Yield of last two steps: 7.5 g (73%).

#### Preparation of ICR-12

A Teflon lined autoclave (Berghof DAB-2) was charged with 10 mg of H_3_PPP(Me) (42 μmol) and 5.7 mg of FeCl_3_·6H_2_O (21 μmol) and overlaid with 10 mL of absolute EtOH. The sealed autoclave was heated in a preheated heating mantle (DAH heating block controlled by BTC-3000 unit) at 120 °C for 24 h. The resulting white powder was centrifuged (11,000 rpm, 5 min, Hettich ROTINA 380 R), washed five times with EtOH (the third time, the powder was left in EtOH for 2 h), three times with water (the second time, the powder was left in water overnight), and three times with acetone (the third time, the powder was left in acetone for 1.5 h) and activated at 80 °C for 5 h under vacuum.

#### Preparation of ICR-13

10 mg of 1,4-phenylenediphosphonic acid (42 μmol) and 5.7 mg of FeCl_3_·6H_2_O (21 μmol) were dry grinded in a ball mill (Laarmann LMLW-320 12) for 30 min (60 Hz). The resulting yellow powder was transferred into a Teflon lined autoclave and overlaid with 5 mL of absolute EtOH. The sealed autoclave was heated in a preheated heating mantle at 250 °C for 24 h. The resulting white powder was centrifuged and washed by the same procedure as ICR-12.

### Instrumental Methods

^1^H and ^31^P NMR spectra were recorded using a JEOL 600 MHz NMR spectrometer. CHN elemental analysis was determined by a standard combustion technique (Thermo Scientific FlashSmartTM 2000Elemental analyzer).

PXRD patterns were recorded using the Debye–Scherrer transmission configuration on the powder diffractometer SmartLab of Rigaku (λ_Cu,Kα1_ = 1.54056 Å) that was equipped by primary monochromator, focusing mirror, capillary holder, and D/tex ultra 250 detector. The sample was ground and placed to the 0.5 mm borosilicate-glass capillary.

Adsorption isotherms of N_2_ at 77 K were recorded using a 3P micro 300 instrument (3P Instruments). Prior to adsorption experiments, the samples were evacuated at 100 °C for at least 24 h.

Thermal analyses (TG/DTA/MS) were carried out on a Setaram SETSYS Evolution-16-MS instrument coupled with a mass-spectrometer. The measurements were performed in synthetic air (flow rate 30 mL min^–1^) from 30 to 750 °C with a heating rate of 5 °C min^–1^.

Fourier transform infrared (FTIR) spectra were recorded with a Nicolet NEXUS 670-FT spectrometer in KBr pellets.

## Supporting Information Available

NMR spectra of the ligands and intermediate products; details concerning the structure determination and refinement; and FTIR, PXRD, TGA, and gas sorption data for ICR-12 and ICR-13 (PDF)
Atomic model of ICR-12 (XYZ)
Atomic model of ICR-13 (XYZ)

## Supplementary Material

ic2c03271_si_001.pdf

ic2c03271_si_002.xyz

ic2c03271_si_003.xyz
