# Supplementary material for: Exploring the Isoreticular Continuum between Phosphonate- and Phosphinate-Based Metal–Organic Frameworks
Source: Inorg Chem. 2022 Nov 11;61(47):18990–7. doi: 10.1021/acs.inorgchem.2c03271 (PMC9709987; doi:10.1021/acs.inorgchem.2c03271)
Supplement: Supplementary file 1 — ic2c03271_si_001.pdf [file ic2c03271_si_001.pdf]

Supporting information

Exploring the isoreticular continuum between phosphonate- and phosphinate-based metal-organic frameworks

Soňa Ondrušová,[a, b] Matouš Kloda,[a] Jan Rohlíček,[c] Marco Taddei,[d] Jan K. Zaręba,[e] Jan Demel[a*]*

[a] Institute of Inorganic Chemistry of the Czech Academy of Sciences, 250 68 Řež, Czech Republic; E-mail: demel@iic.cas.cz

[b] Faculty of Science Charles University, 128 00 Praha 2, Czech Republic

[c] Department of Structure Analysis, Institute of Physics, Czech Academy of Sciences, Prague, 18221, Czech Republic

[d] Department of Chemistry and Industrial Chemistry, University of Pisa, Via Giuseppe Moruzzi, 13, Pisa, 56124, Italy

[e] Institute of Advanced Materials, Wrocław University of Science and Technology, Wybrzeże, Wyspiańskiego 27, Wrocław, 50-370, Poland; E-mail: jan.zareba@pwr.edu.pl

Table of Contents:

Figure S1: ^{1}H NMR spectrum of methyl methylphospinate. 3
Figure S2: ^{31}P NMR spectrum of methyl methylphospinate. 3
Figure S3: ^{1}H NMR spectrum of trimethyl phenylene-1-phosphonate-4-methylphosphinate. 4
Figure S4: ^{31}P NMR spectrum of trimethyl phenylene-1-phosphonate-4-methylphosphinate. 4
Figure S5: ^{1}H NMR spectrum of phenylene-1-phosphonic-4-methyl-phosphinic acid. 5
Figure S6: ^{31}P NMR spectrum of phenylene-1-phosphonic-4-methyl-phosphinic acid. 5
Figure S7: ^{13}C NMR spectrum of phenylene-1-phosphonic-4-methyl-phosphinic acid. 6
Figure S8: The final Rietveld plot of the ICR-12. 7
Figure S9: The final Rietveld plot of the ICR-13. 8
Figure S10: Asymmetric unit of ICR-12. 9
Figure S11: Asymmetric unit of ICR-13. 9
Figure S12: FTIR spectrum of ICR-12. 10
Figure S13: FTIR spectrum of ICR-13. 10
Figure S14: TGA/DTA curves and the evolution of gases for ICR-12 on air. 11
Figure S15: TGA/DTA curves and the evolution of gases for ICR-13 on air. 12

Figure S16: Comparison of PXRD patterns of ICR-2, ICR-12 and ICR-13. 13
Figure S17: PXRD pattern of Al ICR-12 product. 13
Figure S18: PXRD pattern of Al ICR-13 product. 14
Figure S19: Comparison of PXRD patterns of as-synthesized ICR-12 and ICR-12 after treatment with 3M solution of NaOH in EtOH at room temperature. 14
Figure S20: Comparison of PXRD patterns of as-synthesized ICR-13 and ICR-13 after treatment with 3M solution of NaOH in EtOH at room temperature. 15
Figure S21: Pore size distribution of ICR-12 and ICR-13. 15

Table S1: Crystallographic data. 8
Table S2: Coordinates of non-hydrogen atoms in the asymmetric unit of ICR-12. 9
Table S3: Coordinates of non-hydrogen atoms in the asymmetric unit of ICR-13. 9
Table S4: Adsorption and desorption isotherm for ICR-12 16
Table S5: Adsorption and desorption isotherm for ICR-13 17

NMR data

methyl methylphosphinate:

^{1}H NMR ($CDCl_3$): δ 1.55 (dd, $^2J_{PH}$ = 15.1, $^3J_{HH}$ 5.2 Hz, 3H); 3.78 (d, $^3J_{PH}$ = 12.0 Hz, 3H); 7.19 (d, $^1J_{PH}$ = 540 Hz, 1H). ^{31}P{1H} NMR ($CDCl_3$): δ 37.5.

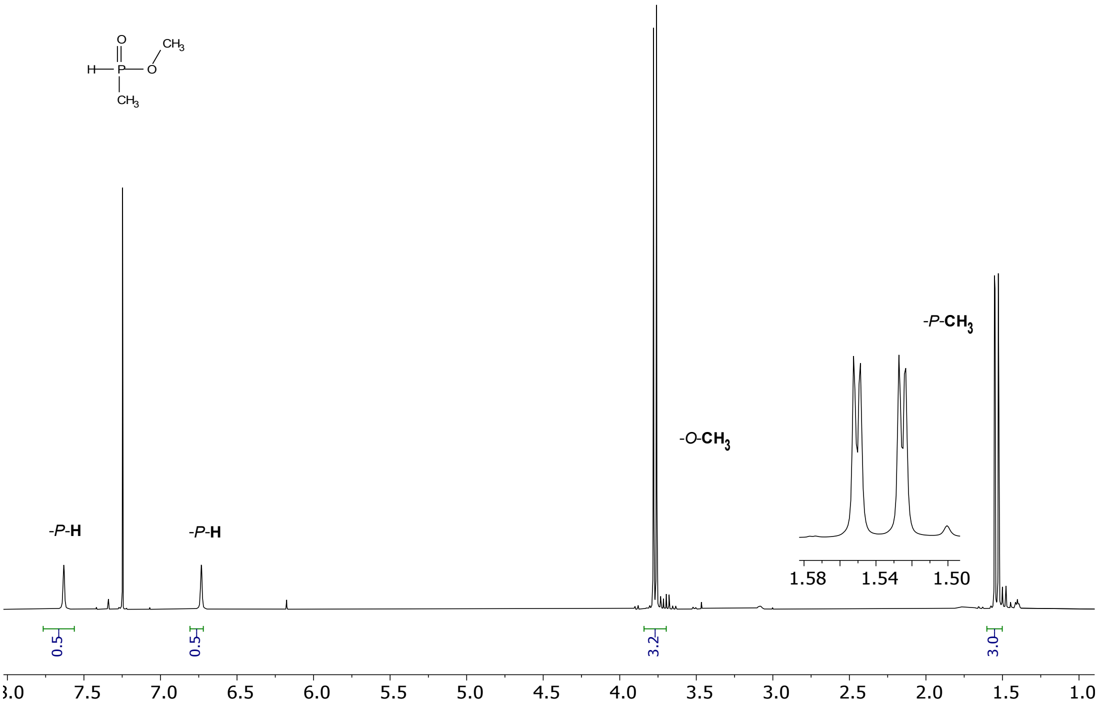

Figure S1: ^{1}H NMR spectrum of methyl methylphospinate.

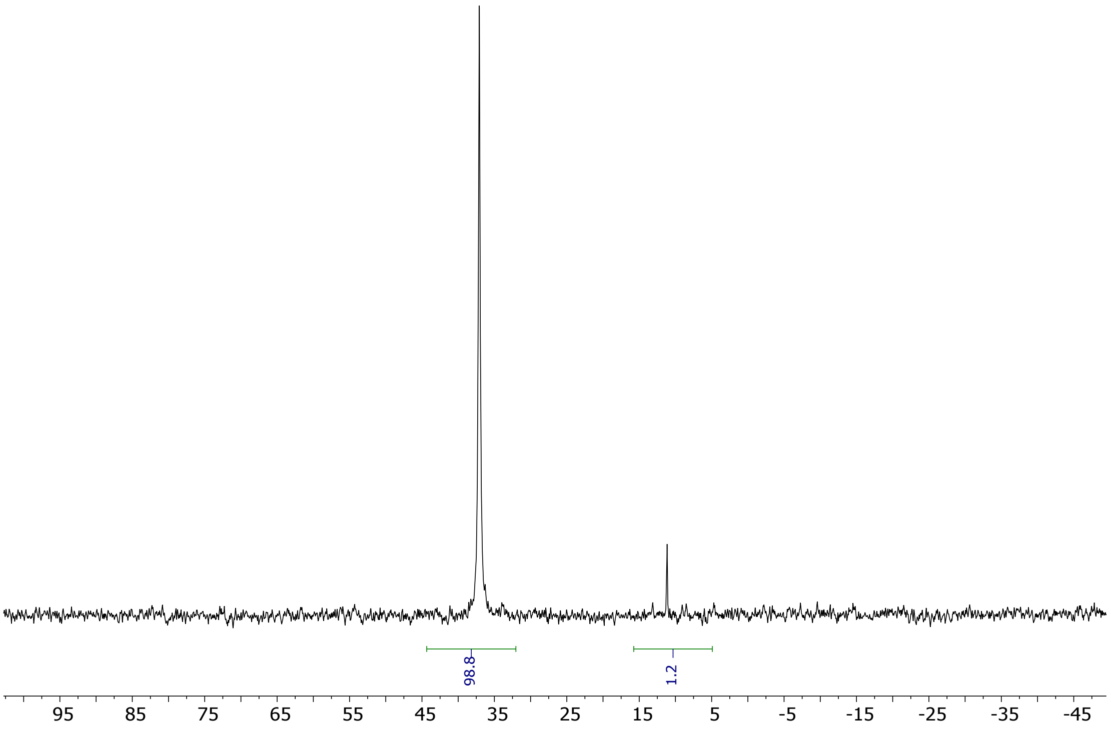

Figure S2: ^{31}P NMR spectrum of methyl methylphospinate.

Diethyl 4-[methoxy(methyl)phosphoryl]phenylphosphonate:

^{1}H NMR ($CDCl_3$): δ 1.33 (t, $^{2}J_{HH}$ = 7.0 Hz, 6H); 1.68 (d, $^{2}J_{PH}$ = 14.5 Hz, 3H); 3.62 (d, $^{3}J_{PH}$ = 11.4 Hz); 4.14 (m, 4H); 7.9 (m, 4H). ^{31}P{1H} NMR ($CDCl_3$): δ 17.37 (s, 1H); 43.46 (s, 1H).

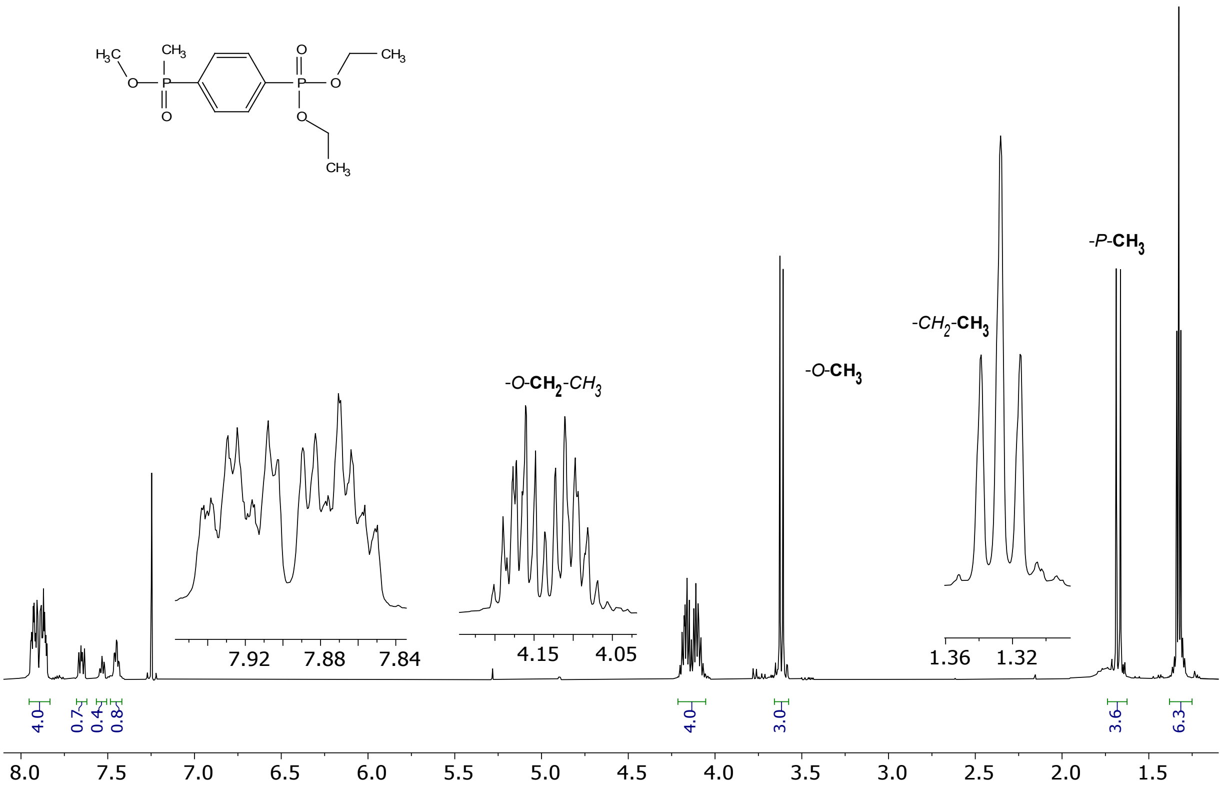

Figure S3: ^{1}H NMR spectrum of trimethyl phenylene-1-phosphonate-4-methylphosphinate.

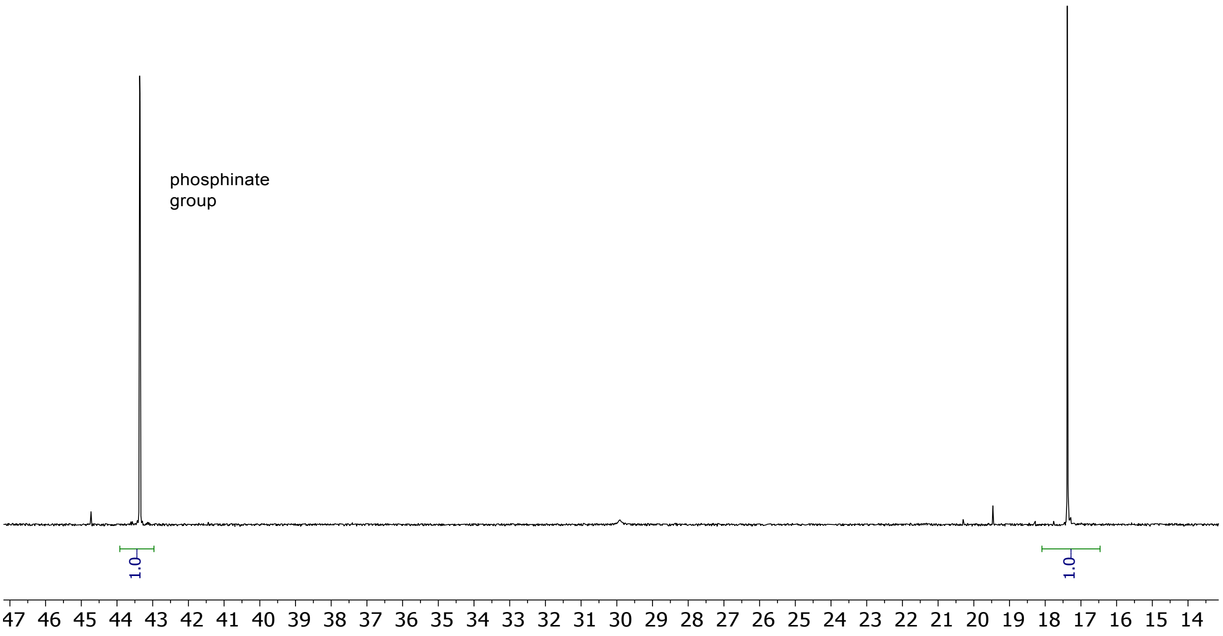

Figure S4: ^{31}P NMR spectrum of trimethyl phenylene-1-phosphonate-4-methylphosphinate.

4-[hydroxy(methyl)phosphoryl]phenylphosphonic acid:

^{1}H NMR (MeOD): δ 1.65(d, $^{2}J_{HH}$ = 14.7, 3H); 7.90 (m, 4H). ^{31}P{1H} NMR (MeOD): δ 14.63 (s, 1H,); 39.51 (s, 1H). ^{13}C{1H} NMR(MeOD): δ 15.02 (d, J_{PC} = 101.2 Hz); δ 130.19 (dd, J = 14.6, 10.4 Hz); δ 130.65 (dd, $^{1}J_{PC}$ = 12.7, 10.0 Hz); δ 136.10 (dd, J_{PC} = 84.7, 2.2 Hz); δ 137.14 (dd, J_{PC} = 143.0, 2.9 Hz).

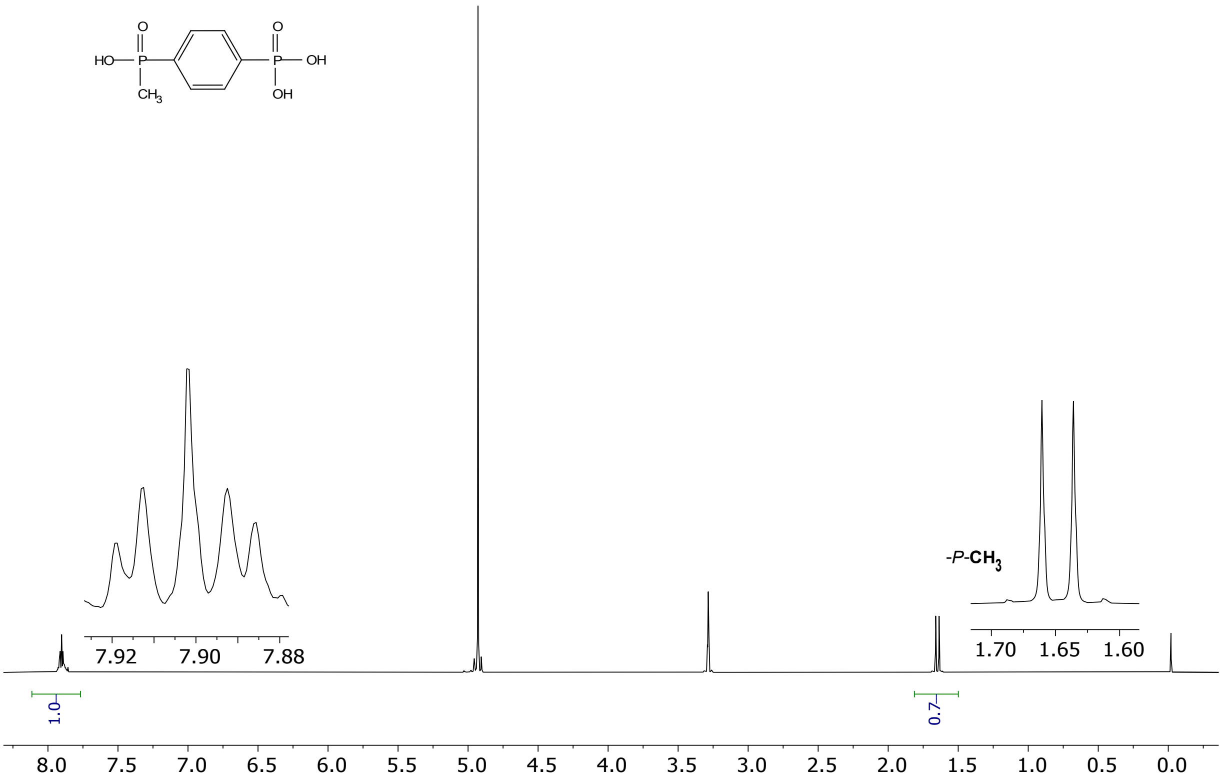

Figure S5: ^{1}H NMR spectrum of phenylene-1-phosphonic-4-methyl-phosphinic acid.

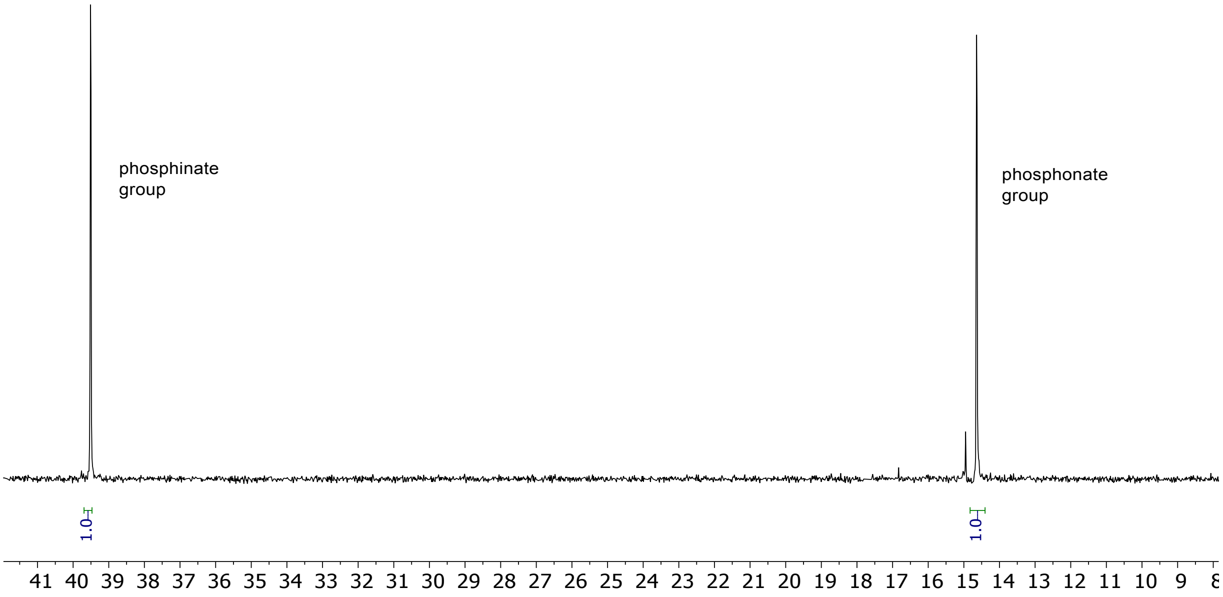

Figure S6: ^{31}P NMR spectrum of phenylene-1-phosphonic-4-methyl-phosphinic acid.

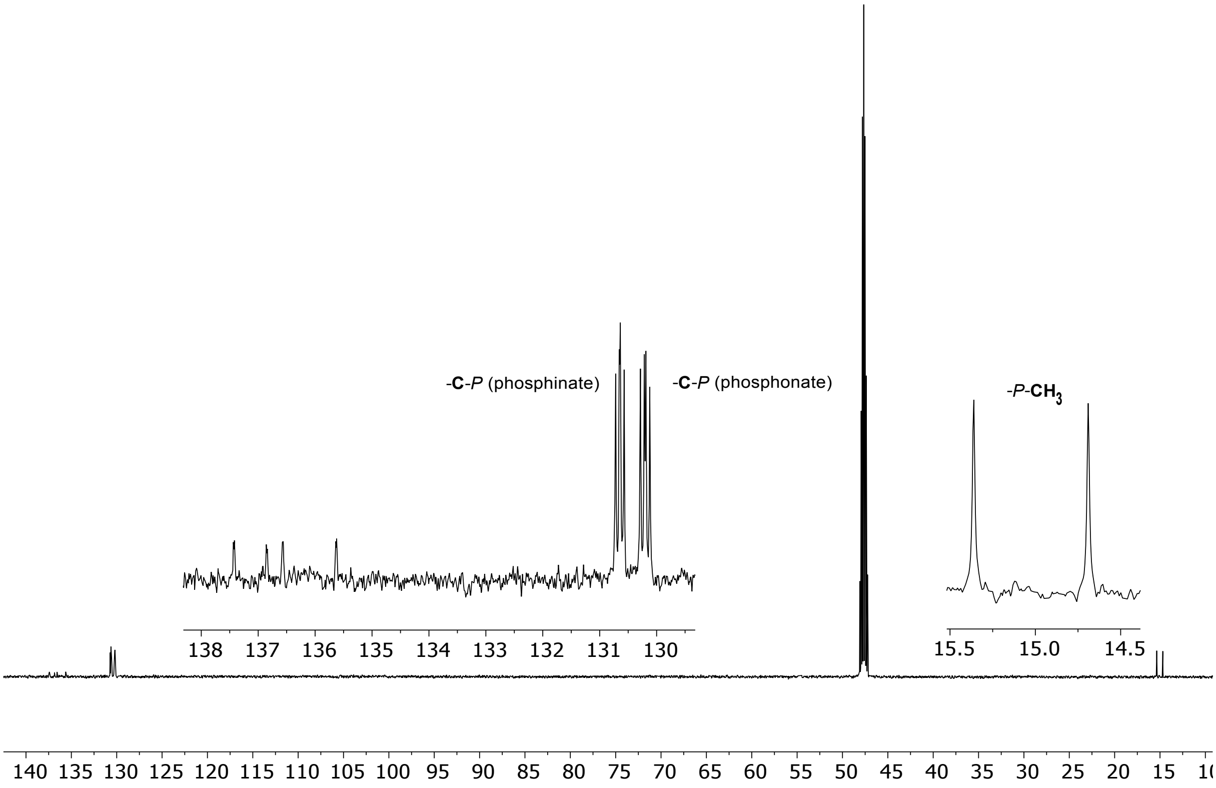

Figure S7: ^{13}C NMR spectrum of phenylene-1-phosphonic-4-methyl-phosphinic acid.

Structure determination

The indexing of the ICR-12 and ICR-13 PXRD patterns in the program DICVOL[1] revealed the possible trigonal unit cells that are similar to each other and that are similar to already known related compound ICR-4, see Table S1 with crystallographic data of ICR-12 and ICR-13.

Since the quality of the measured data of compound ICR-12 was not high enough to resolve the structure, a crystal structure model was derived from ICR-4 in order to confirm the possible honeycomb arrangement and thus the similarity to other ICR compounds. Although the final Rietveld fit shows mainly agreement between calculated and measured data, there are several very poorly fitted diffractions in the record. This suggests that the current crystal structure model does not adequately describe the true model, and some parts of the crystal structure may be missing or wrong. Unfortunately, the low quality of the measured pattern does not allow to calculate the difference Fourier with sufficient resolution to see more details of the missing crystal structure. The presence of a strong maximum indicates missing atoms in the centre of the pore. Placing the Fe atom in this position led to a significant decrease in the R profile values and an increase in the occupation factor of the placed Fe atom to an unrealistic occ ~ 2.5, see Rietveld fit in Fig. S1.

The crystal structure of ICR-13 was partially solved in Superflip[2] and then gradually completed by a combination of Rietveld refinement and manual placement of fragments into a difference Fourier map in MCE[3] . The crystal structure of ICR-13 exhibits a honeycomb arrangement analogous to ICR-4 or ICR-2. The final Rietveld refinement in program Jana2020[4] did not result in a satisfactory profile fit. The difference Fourier map revealed an additional electron density in the pores that could not be modelled by any reasonable molecular or atomic model, see Figure 3. For example, the residual electron density

in the pores can be modelled reasonably well by the partial occupancy of oxygen atoms in the pore centres and at positions approximately 2 Å from the oxygen atoms of the phosphonate groups, where the occupancies are approximately occ = 1.1 and occ = 1.7 and the atomic displacement parameters (ADPs) are extremely high: U_{iso} ~ 0.4 Å^2, see Rietveld fit in Fig. S2. Both occupancy factors and high ADPs show that this atomic model made by oxygen atoms is not correct. Nevertheless, the high ADPs blurred the electron density of the placed oxygen atoms and allowed a relatively good approximation of the electron density in the pores, leading to a significant reduction of the profile R factors and resulting in a satisfactory profile fit. Similarly good profile fit can also be achieved by placing partially occupied phenyls or Fe atoms in the residual electron density. Although the structure in the pores remains unclear, the honeycomb arrangement of the main structural motif was confirmed.

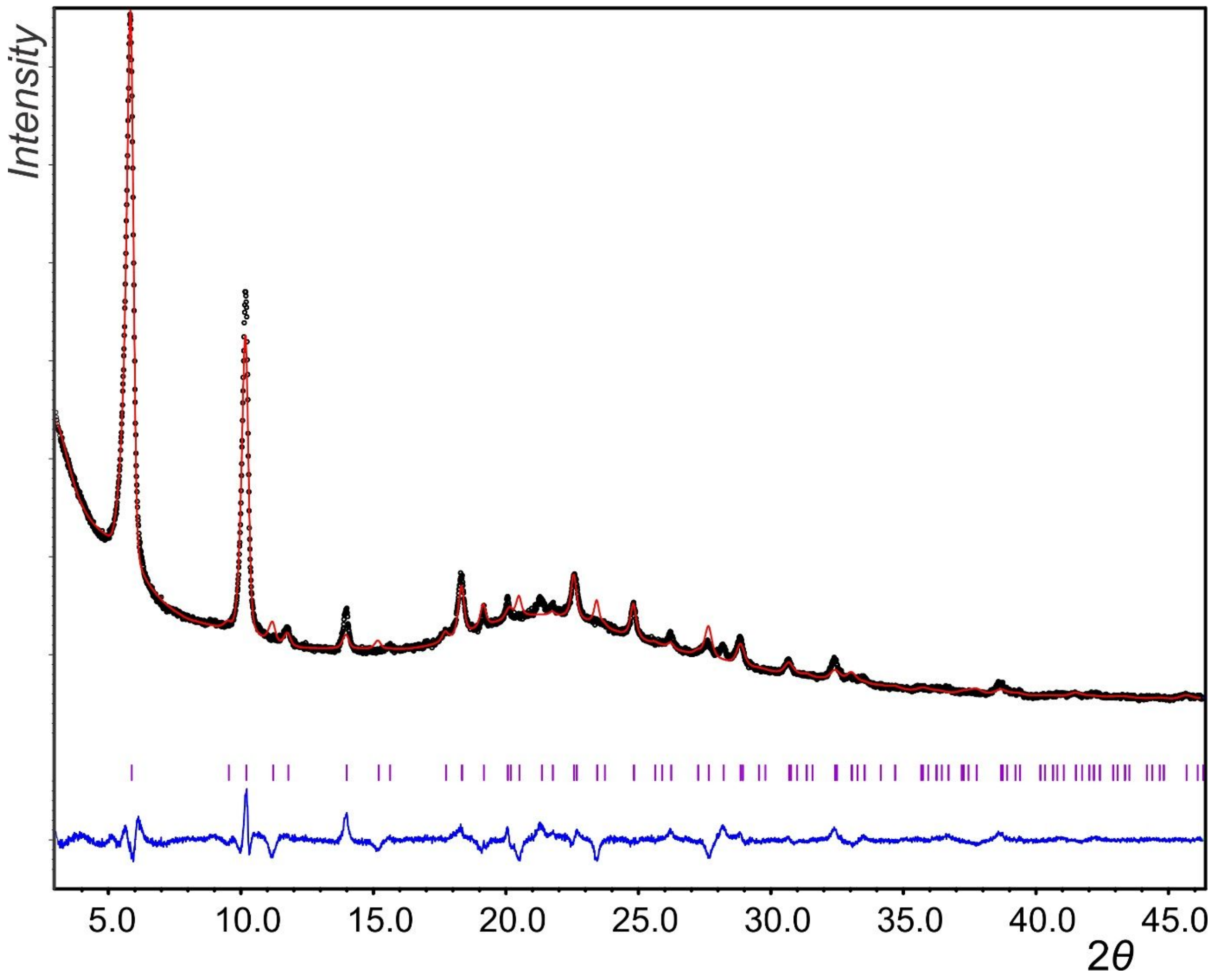

Figure S8: The final Rietveld plot of the ICR-12 sample shows significant discrepancies between the measured and calculated profiles. This is mainly because the structural model does not describe the residual el. denisty inside the pore. The XRPD pattern was measured to 80° 2θ, but only the part with observable diffraction peaks is shown. Black dots – measured points, red – calculated profile, magenta bars – Bragg's positions, blue – difference curve.

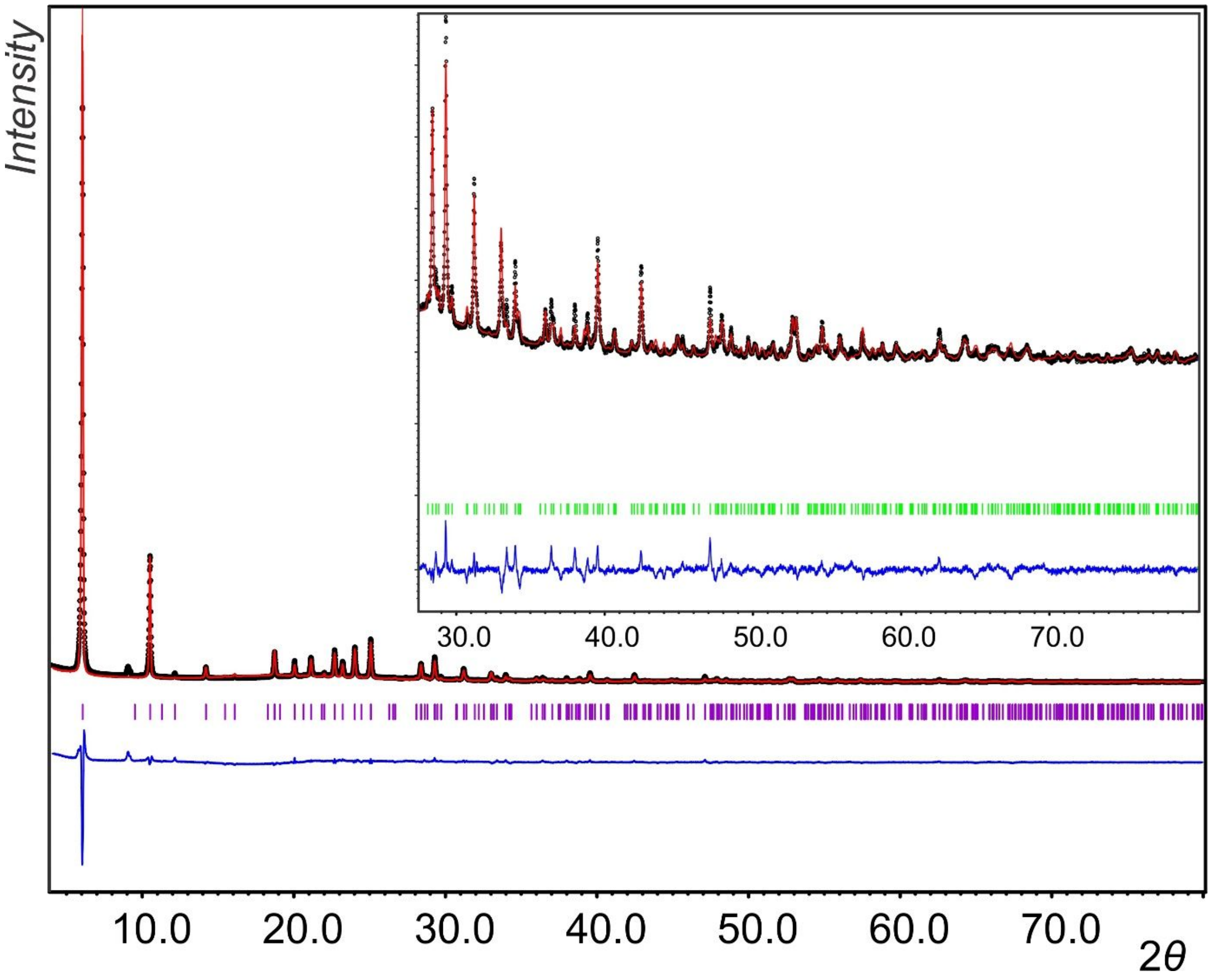

Figure S9: The final Rietveld plot of the ICR-13 sample shows relatively good agreement with the calculated profile of the structural model even if the structural model inside the pore does not properly describe the real structure. Black dots – measured points, red – calculated profile, magenta bars – Bragg's positions, blue – difference curve.

Table S1: Crystallographic data.

	Fe-ICR-12	Fe-ICR-13
Formula	$C_{48} H_{60} Fe_5 O_{36} P_{18}$	$C_9 H_6 Fe O_9 P_3$
a	17.2944	16.7958
c	9.2496	9.2870
Volume	2395.874	2268.859
Crystal system	trigonal	trigonal
Space group	*P*3	*P*3
R_p	0.1620	0.0836
R_{wp}	0.1804	0.1036
GOF	8.1386	7.71

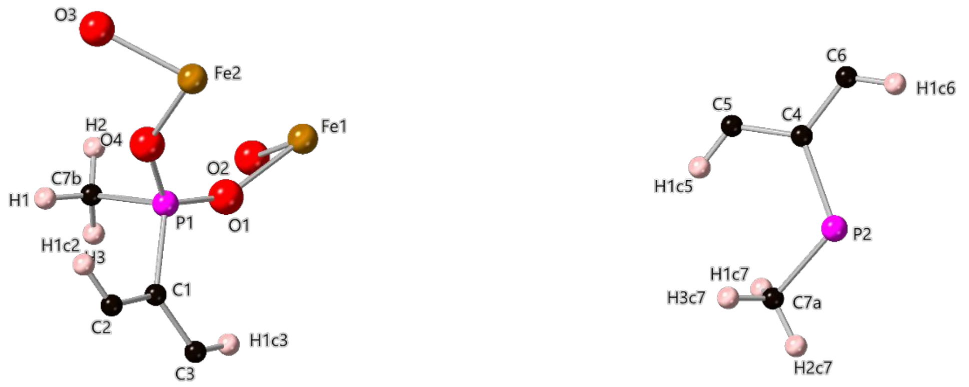

Figure S10: Asymmetric unit of ICR-12. The quality of available data was not sufficient to differentiate between -OH and $-CH_3$ of the ligand and only $-CH_3$ was used in the model.

Table S2: Coordinates of non-hydrogen atoms in the asymmetric unit of ICR-12.

Atom	x	y	z	Atom	x	y	z
C1	0.5687	0.084	0.5277	O1	0.6473	0.2328	0.6965
C2	0.5594	0.0487	0.3974	O2	0.7401	0.3051	0.9456
C3	0.5089	0.0362	0.6299	O3	0.768	0.3545	0.1915
C4	-0.082	0.4275	0.0254	O4	0.6875	0.2513	0.4307
C5	-0.0099	0.417	0.006	P1	0.6563	0.1966	0.5622
C6	-0.0729	0.5097	0.017	P2	-0.1912	0.3307	0.058
C7a	-0.1846	0.2326	0.0955	Fe1	2/3	1/3	0.8188
C7b	0.7514	0.1879	0.603	Fe2	2/3	1/3	0.3133

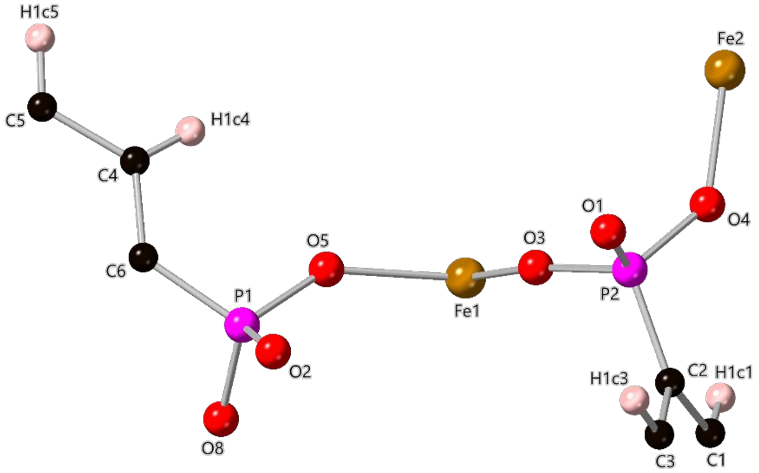

Figure S11: Asymmetric unit of ICR-13.

Table S3: Coordinates of non-hydrogen atoms in the asymmetric unit of ICR-13.

Atom	x	y	z	Atom	x	y	z
C1	0.073	0.484	0.528	O3	0.251	0.687	0.487
C2	0.085	0.572	0.557	O4	0.218	0.628	0.736
C3	0.011	0.587	0.531	O5	0.371	0.776	0.228
C4	0.516	0.957	0.118	O8	0.314	0.750	-0.022
C5	0.587	1.046	0.067	P1	0.343	0.816	0.104
C6	0.428	0.914	0.056	P2	0.182	0.657	0.610
O1	0.168	0.736	0.656	Fe1	1/3	2/3	0.355
O2	0.262	0.828	0.152	Fe2	1/3	2/3	0.851

FTIR spectra

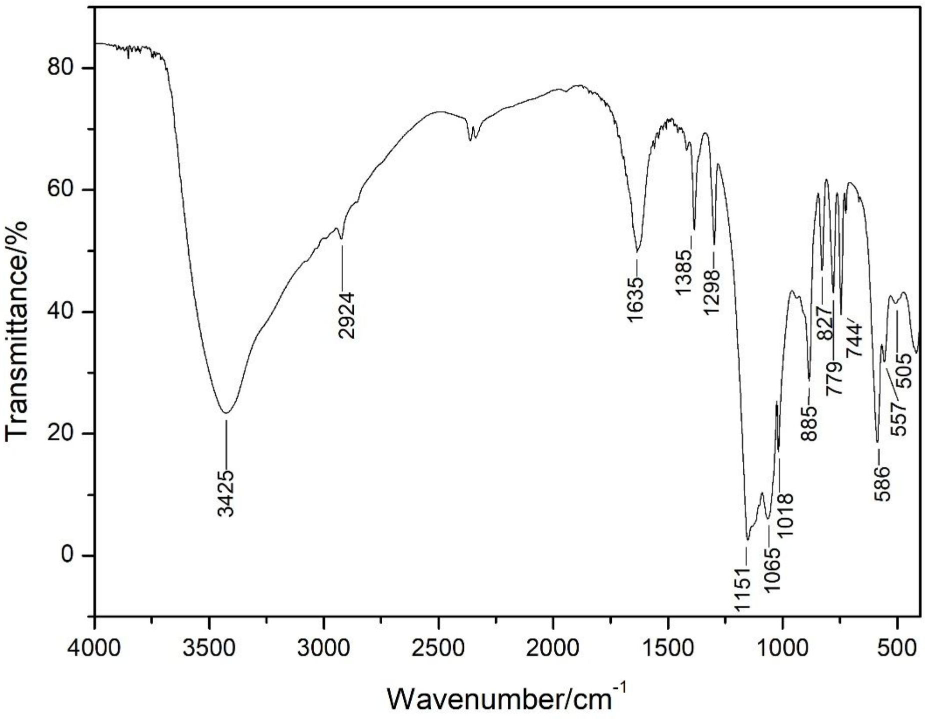

Figure S12: FTIR spectrum of ICR-12.

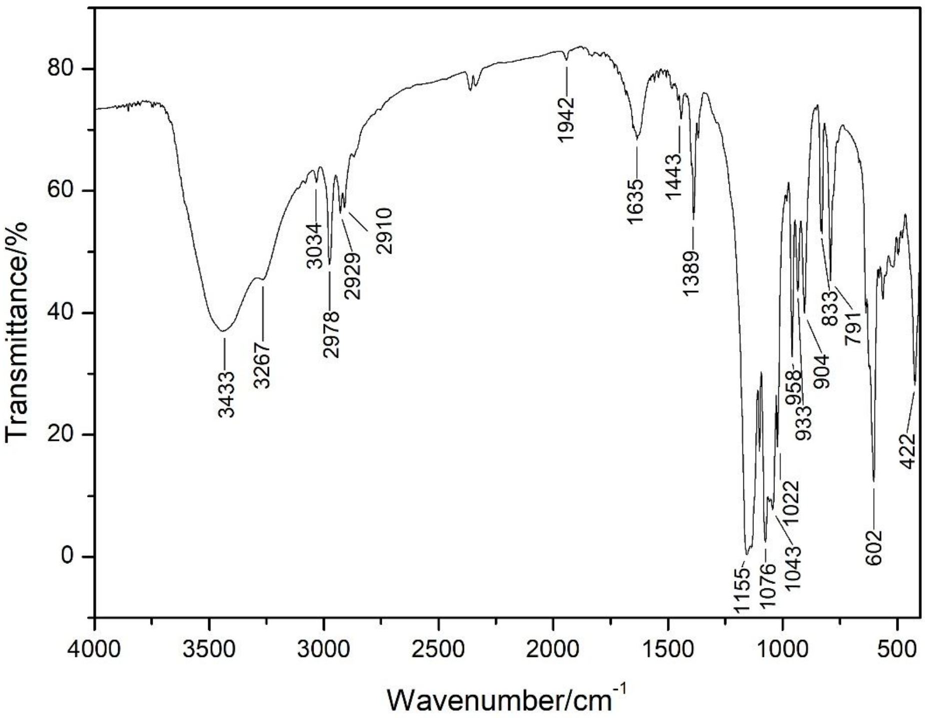

Figure S13: FTIR spectrum of ICR-13.

TG/DTA results

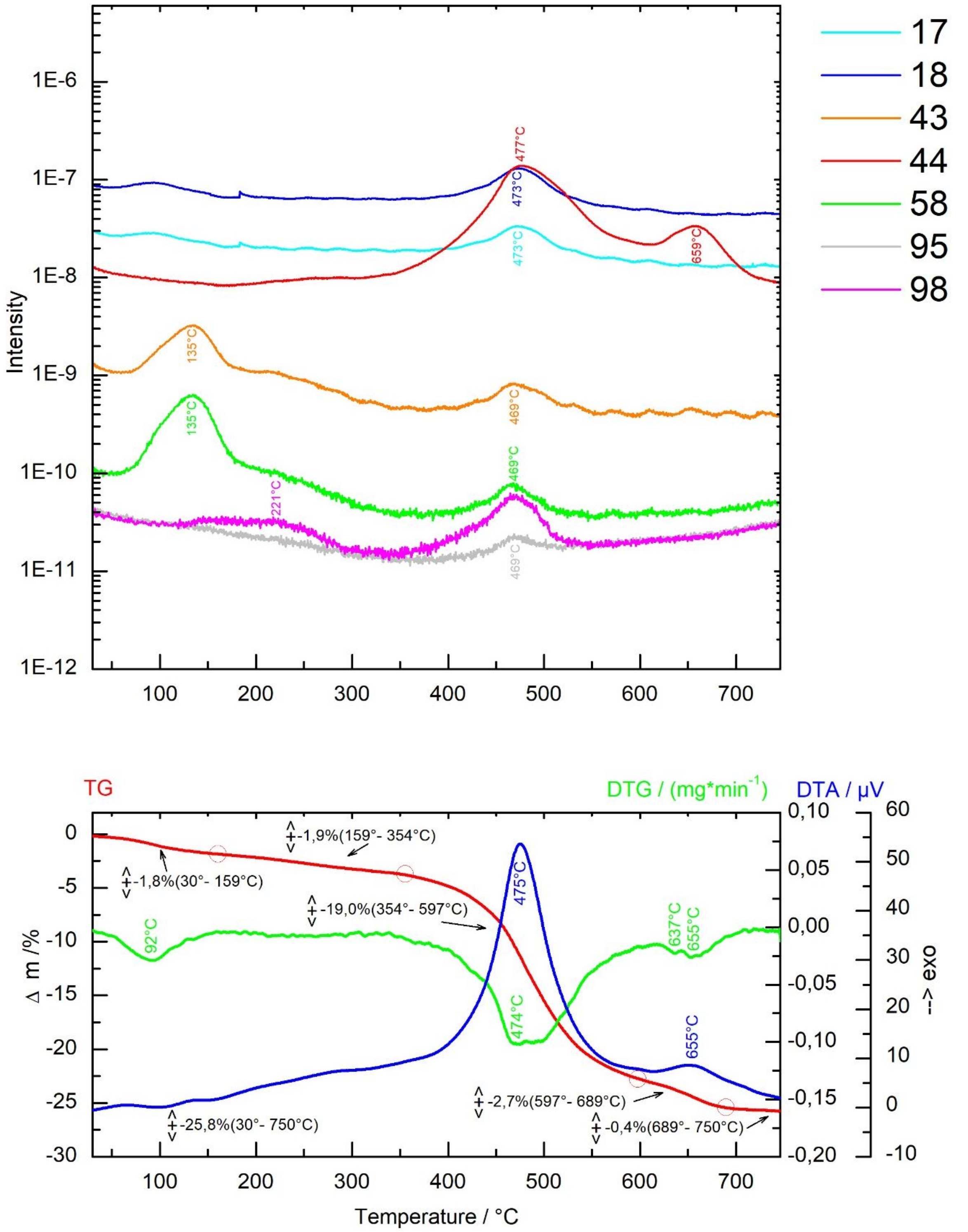

Figure S14: TGA/DTA curves and the evolution of gases for ICR-12 on air; m/z = 17 – OH, m/z = 18 – H_2O, m/z = 44 – CO_2, m/z = 43 and 58 – acetone, and m/z = 98 – H_3PO_4.

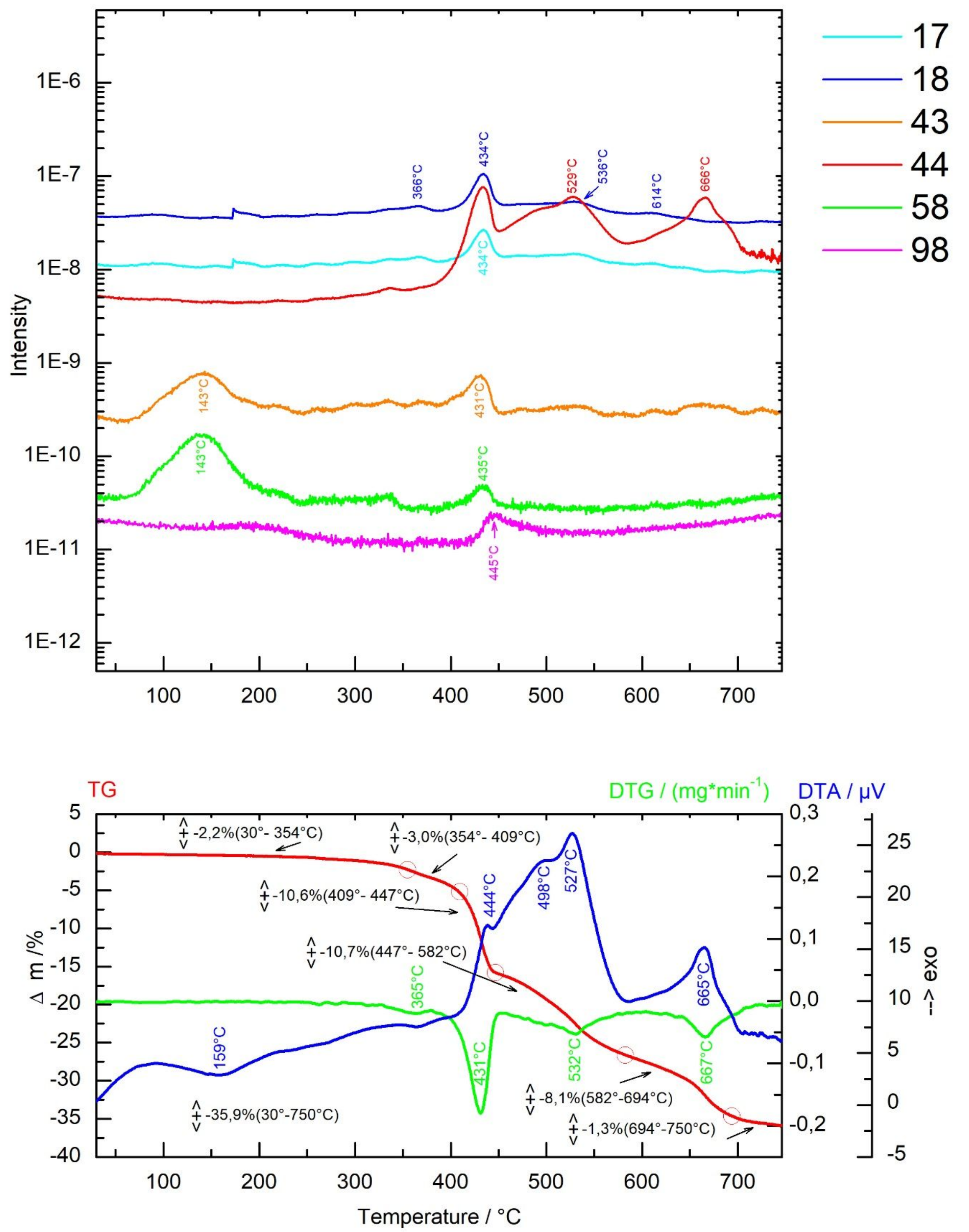

Figure S15: TGA/DTA curves and the evolution of gases for ICR-13 on air; m/z = 17 – OH, m/z = 18 – H_2O, m/z = 44 – CO_2, m/z = 43 and 58 – acetone, and m/z = 98 – H_3PO_4.

PXRD patterns

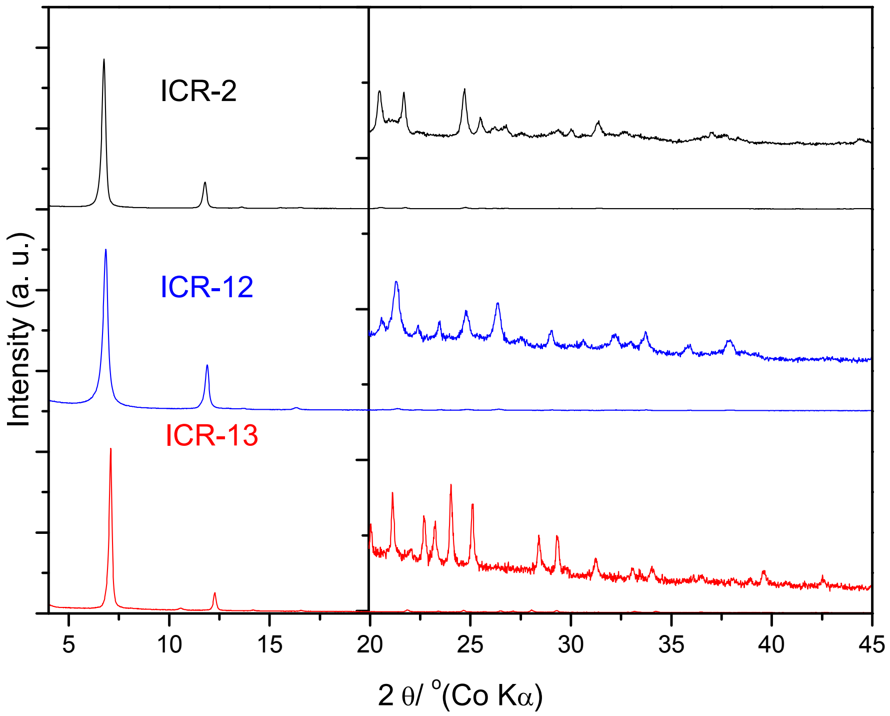

Figure S16: Comparison of PXRD patterns of ICR-2, ICR-12 and ICR-13. The diffractograms are vertically shifted to avoid overlaps.

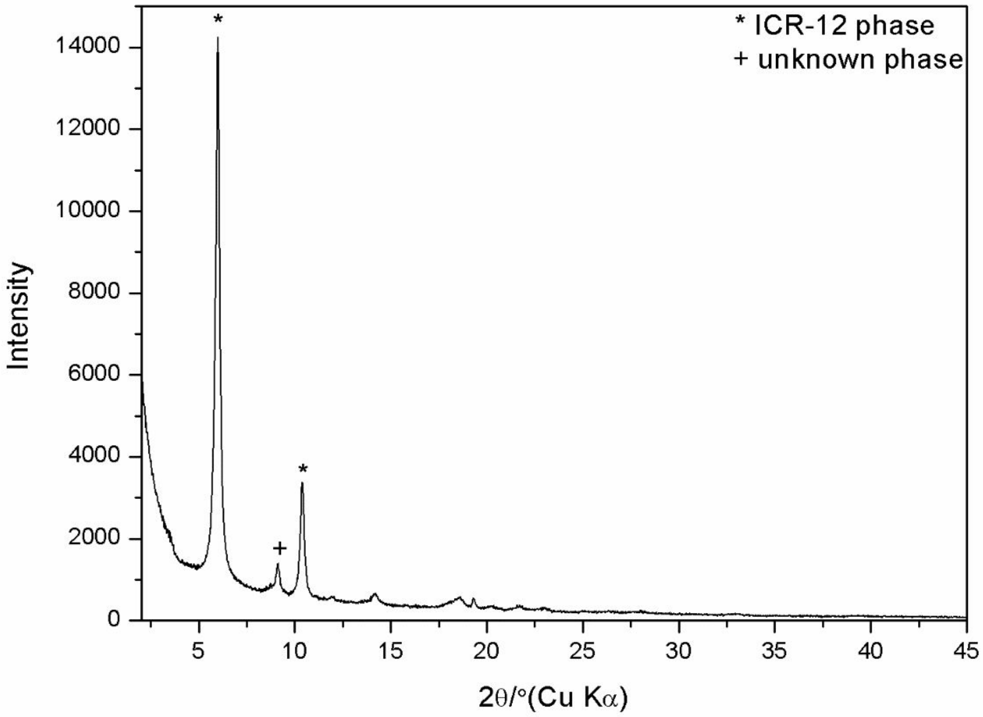

Figure S17: PXRD pattern of Al ICR-12 product. * is ICR-12 phase, + is unknown phase.

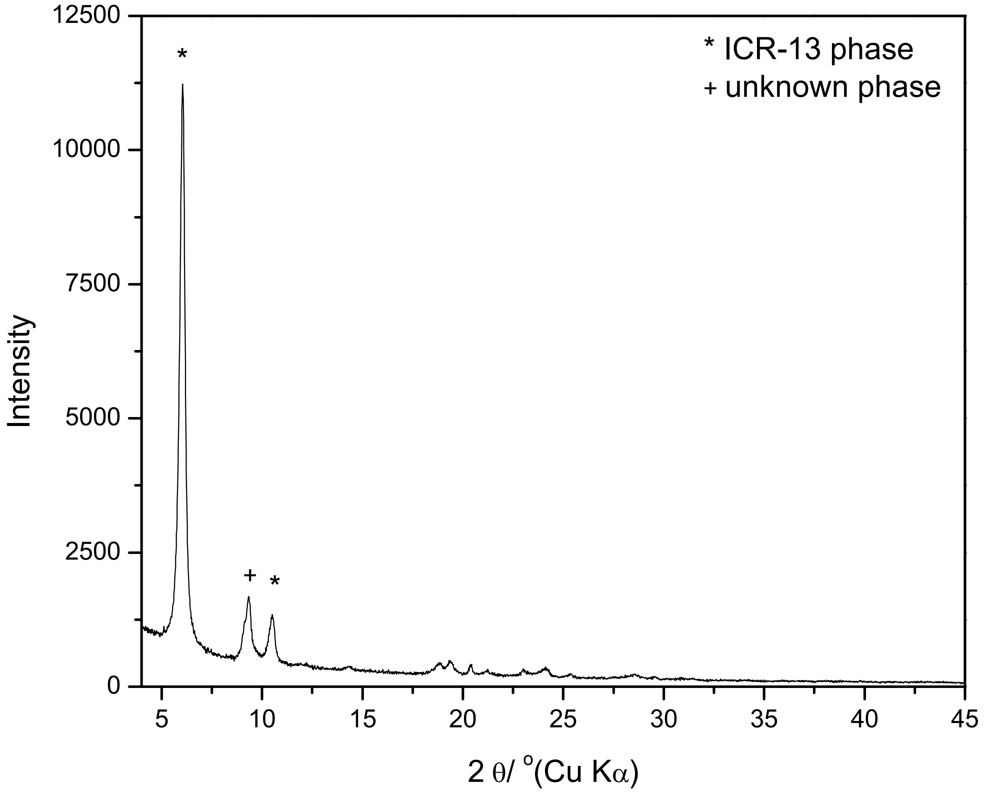

Figure S18: PXRD pattern of Al ICR-13 product. * is ICR-13 phase, + is unknown phase.

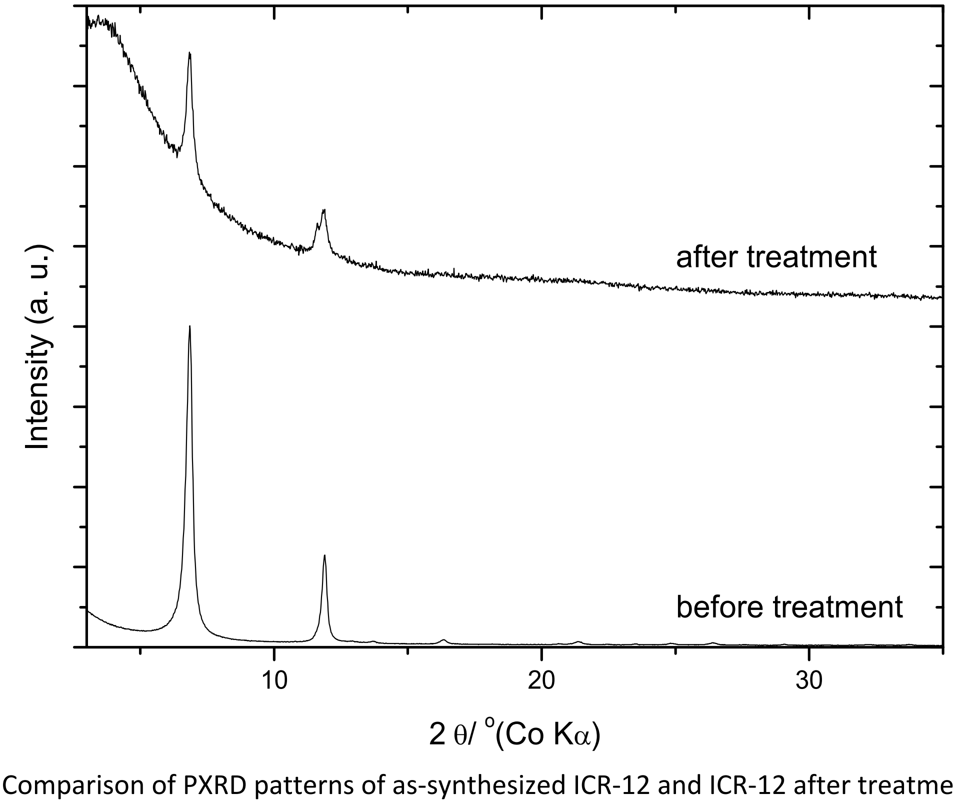

Figure S19: Comparison of PXRD patterns of as-synthesized ICR-12 and ICR-12 after treatment with 3M solution of NaOH in EtOH at room temperature. The diffractograms are vertically shifted to avoid overlaps.

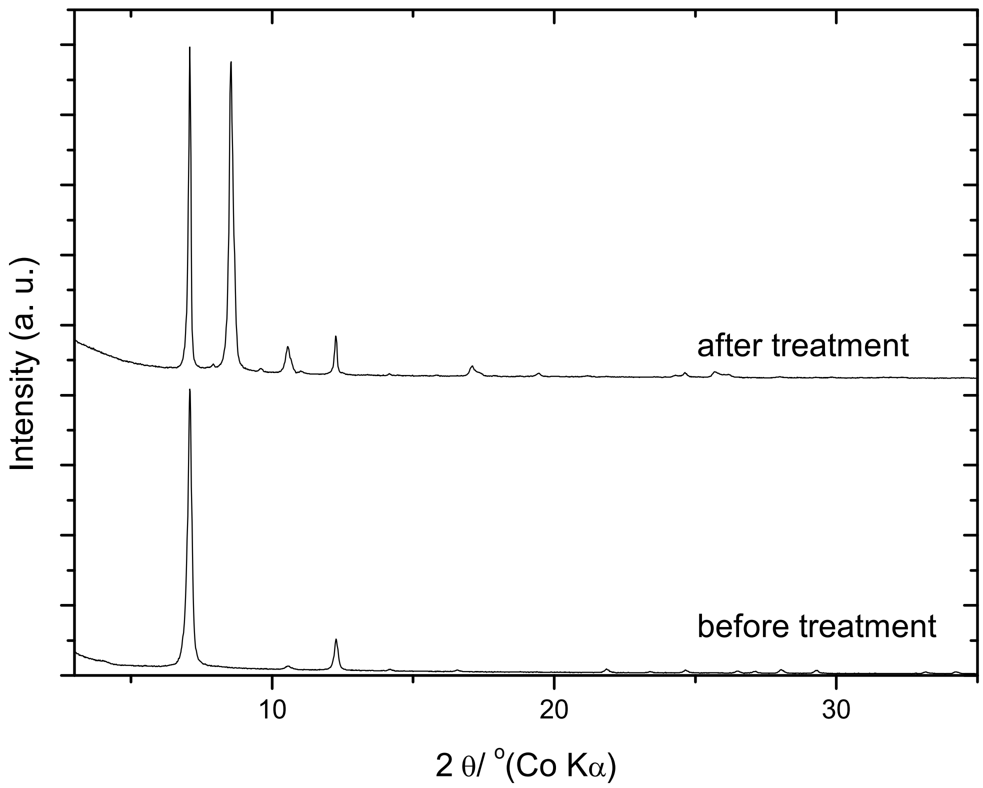

Figure S20: Comparison of PXRD patterns of as-synthesized ICR-13 and ICR-13 after treatment with 3M solution of NaOH in EtOH at room temperature. The diffractograms are vertically shifted to avoid overlaps.

Gas sorption results

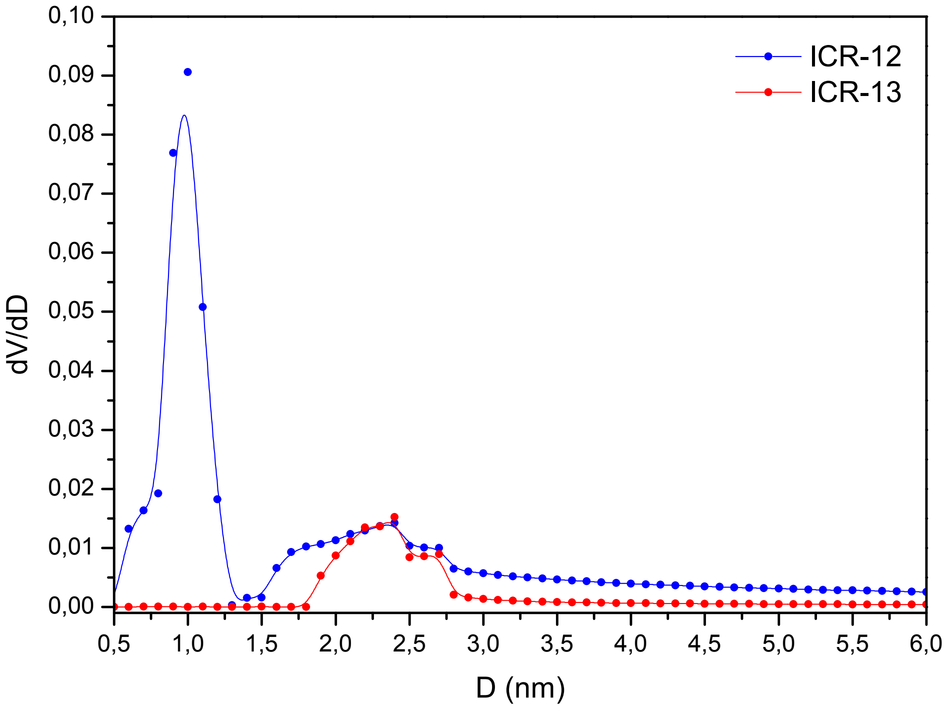

Figure S21: Pore size distribution of ICR-12 and ICR-13.

Table S4: Adsorption and desorption isotherm for ICR-12

**Adsorption**		**Desorption**	
P/P_0	V_{ads} (cm^3g^{-1} STP)	P/P_0	V_{ads} (cm^3g^{-1} STP)
$3{,}98 \cdot 10^{-5}$	1,76208	0,992911	374,09355
$4{,}76 \cdot 10^{-5}$	10,7497	0,96512	343,12722
0,000121	19,67909	0,949197	302,54851
0,000268	28,5619	0,933153	262,1616
0,00045	37,44089	0,910383	227,2519
0,000718	46,25814	0,878817	200,25257
0,001222	54,85299	0,837814	181,2422
0,002047	63,21293	0,787674	167,76324
0,00328	71,27926	0,734804	158,08079
0,005324	78,74498	0,680568	150,42868
0,010612	83,89245	0,62567	143,94305
0,053975	94,617	0,570169	138,27316
0,107267	100,65956	0,513684	133,25078
0,216983	109,38233	0,457119	127,94407
0,326539	117,06881	0,401176	123,35425
0,435292	124,7929	0,332463	118,33259
0,541535	133,27609	0,271962	113,9217
0,639846	142,65491	0,21353	109,56642
0,750165	156,90111	0,157366	105,19554
0,853818	180,2931	0,10313	100,38447
0,926527	225,77246	0,081263	97,71114
0,958835	300,90964		
0,992911	374,09355		

Table S5: Adsorption and desorption isotherm for ICR-13

Adsorption		Desorption	
P/P_0	V_{ads} (cm^3g^{-1} STP)	P/P_0	V_{ads} (cm^3g^{-1} STP)
0,003137	0,72272	0,991325	616,0831
0,015341	5,58968	0,989859	565,7472
0,050943	7,77032	0,987349	520,1853
0,08556	9,37795	0,98506	473,94
0,127166	12,04811	0,982409	426,9577
0,202064	14,59142	0,980261	377,3246
0,276079	18,40096	0,978654	327,6533
0,349993	21,91917	0,97508	281,1046
0,422321	25,32864	0,971606	234,4861
0,494929	29,12399	0,967269	190,934
0,571735	32,99455	0,961245	147,5249
0,642296	37,01055	0,953093	105,4333
0,711913	41,20374	0,935462	76,22275
0,780225	45,99229	0,905584	64,66195
0,859601	53,59484	0,870464	56,20939
0,931848	70,98325	0,833396	51,08502
0,973735	120,3157	0,797353	47,63281
0,980984	215,0362	0,75884	44,49323
0,98502	314,943	0,720226	41,78648
0,98761	411,6882	0,682014	39,40286
0,989759	515,2107	0,644485	37,14238
0,991325	616,0831	0,607277	35,01741
		0,57051	32,93282
		0,534166	31,10126
		0,49748	29,2525
		0,461255	27,36381
		0,424971	25,46719
		0,387984	23,71004
		0,351298	21,9807
		0,304612	19,75912
		0,26385	17,77155
		0,223148	15,67661
		0,184715	14,0023
		0,148049	12,75724
		0,111363	11,03391
		0,074617	8,86974

[1] Boultif, A.; Louër, D. Powder Pattern Indexing with the Dichotomy Method. *J. Appl. Cryst.*, **2004**, *37*, 724–731. https://doi.org/10.1107/s0021889804014876.

[2] Palatinus, L.; Chapuis, G. SUPERFLIP– a Computer Program for the Solution of Crystal Structures by Charge Flipping in Arbitrary Dimensions. *J. Appl. Cryst.*, **2007**, *40*, 786–790. https://doi.org/10.1107/s0021889807029238.

[3] Rohlíček, J.; Hušák, M. MCE2005– a New Version of a Program for Fast Interactive Visualization of Electron and Similar Density Maps Optimized for Small Molecules. *J. Appl. Cryst.*, **2007**, *40*, 600–601. https://doi.org/10.1107/s0021889807018894.

[4] Petříček, V.; Dušek, M.; Palatinus, L. Crystallographic Computing System JANA2006: General Features. *Z. Kristallogr. Cryst. Mater.*, **2014**, *229*, 345–352. https://doi.org/10.1515/zkri-2014-1737.
